# A New Subclass of Exoribonuclease-Resistant RNA Found in Multiple Genera of *Flaviviridae*

**DOI:** 10.1128/mBio.02352-20

**Published:** 2020-09-29

**Authors:** Matthew J. Szucs, Parker J. Nichols, Rachel A. Jones, Quentin Vicens, Jeffrey S. Kieft

**Affiliations:** a Department of Biochemistry and Molecular Genetics, University of Colorado Denver School of Medicine, Aurora, Colorado, USA; b RNA BioScience Initiative, University of Colorado Denver School of Medicine, Aurora, Colorado, USA; Washington University School of Medicine

**Keywords:** RNA structure, chemical probing, exoribonuclease-resistant RNAs, subgenomic flavivirus RNAs, viral RNA

## Abstract

Viruses have developed innovative strategies to exploit the cellular machinery and overcome the antiviral defenses of the host, often using specifically structured RNA elements. Examples are found in the *Flavivirus* genus (in the family *Flaviviridae*), where during flaviviral infection, pathogenic subgenomic flaviviral RNAs (sfRNAs) accumulate in the cell. These sfRNAs are formed when a host cell 5′ to 3′ exoribonuclease degrades the viral genomic RNA but is blocked by an exoribonuclease-resistant RNA structure (xrRNA) located in the viral genome’s 3′ untranslated region (UTR). Although known to exist in several *Flaviviridae* genera, the full distribution and diversity of xrRNAs in this family were unknown. Using the recently solved high-resolution structure of an xrRNA from the divergent flavivirus Tamana bat virus (TABV) as a reference, we used bioinformatic searches to identify xrRNAs in the remaining three genera of *Flaviviridae*: *Pegivirus*, *Pestivirus*, and *Hepacivirus*. We biochemically and structurally characterized several examples, determining that they are genuine xrRNAs with a conserved fold. These new xrRNAs look superficially similar to the previously described xrRNAs but possess structural differences making them distinct from previous classes of xrRNAs. Overall, we have identified the presence of xrRNA in all four genera of *Flaviviridae*, but not in all species. Our findings thus require adjustments of previous xrRNA classification schemes and expand the previously known distribution of xrRNA in *Flaviviridae.*

## Abstract (precis)

The members of the *Flaviviridae* comprise one of the largest families of positive-sense single-stranded RNA (+ssRNA) and are divided into the *Flavivirus*, *Pestivirus*, *Pegivirus*, and *Hepacivirus* genera. The genus *Flavivirus* contains many medically relevant viruses such as Zika virus, dengue virus, and Powassan virus. In these, a part of the RNA of the virus twists up into a distinct three-dimensional shape called an exoribonuclease-resistant RNA (xrRNA) that blocks the ability of the cell to “chew up” the viral RNA. Hence, part of the RNA of the virus remains intact, and this protected part is important for viral infection. These xrRNAs were known to occur in flaviviruses, but whether they existed in the other members of the family was not known. In this study, we identified a new subclass of xrRNA found not only in flaviviruses but also in the remaining three genera. The fact that these structured viral RNAs exist throughout the *Flaviviridae* family suggests they are important parts of the infection strategy of diverse pathogens, which could lead to new avenues of research.

## INTRODUCTION

Viruses face continuous evolutionary pressure to evolve innovative strategies that exploit the host cell’s biological machinery and overcome its antiviral defenses. Often these are based on specifically structured RNA elements, which is not surprising given RNA’s functional diversity and ability to influence virtually every cellular process. Important examples are found in positive-sense single-stranded RNA (+ssRNA) viruses such as arthropod-borne flaviviruses, which use several structured elements within their RNA genome to direct or regulate important processes during infection (1–13).

**FIG 1:**
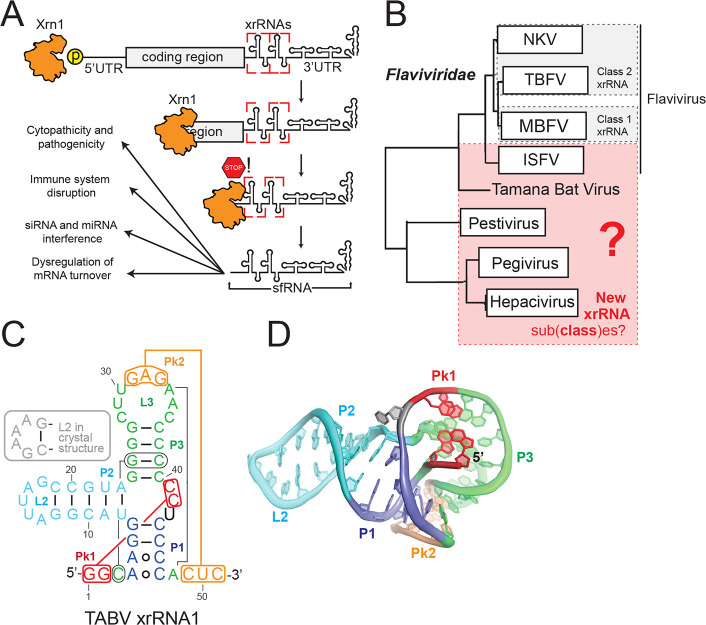
Mechanism and presence of exoribonuclease-resistant RNA in *Flaviviridae*. (A) Mechanism of sfRNA biogenesis mediated through Xrn1 exoribonuclease (orange)-resistant RNA (xrRNA, boxed in red). siRNA, small interfering RNA; miRNA, microRNA. (B) Phylogeny of *Flaviviridae* based on the sequence of NS5 adapted from a previous study (44). Genera possessing class 1 or class 2 xrRNAs are boxed in gray. Genera investigated in this study are boxed in red. NKV, no-known-vector flavivirus; TBFV, tick-borne flavivirus; MBFV, mosquito-borne flavivirus. (C) Secondary structure diagram of the xrRNA1 from TABV. Secondary structure regions are delineated by different colors, and key long-range interactions are indicated with lines. (D) Three-dimensional structure of TABV xrRNA xr1, colored as indicated in panel C (PDB identifier [ID] 7K16) (37).

Among the important structured RNA elements found in flaviviruses are exoribonuclease-resistant RNAs (xrRNAs), which enable an elegant mechanism of noncoding RNA biogenesis. Originally identified in mosquito-borne flaviviruses (MBFVs) (14–19) and later in +ssRNA plant-infecting viruses (20, 21), xrRNAs block the processive degradation of the viral genome by host cell 5′–3′ exoribonuclease Xrn1 (19), the enzyme responsible for the majority of cytoplasmic RNA decay (Fig. 1A) (22). During infection, a subset of flaviviral genomes is targeted to the cellular RNA decay machinery and then processively degraded in a 5′ to 3′ direction by Xrn1 until the enzyme halts at a defined location at the xrRNA in the 3′ untranslated region (UTR) (19, 23). The fold of the xrRNA is sufficient for this function, and no accessory proteins or chemical modifications of the RNA are required. The xrRNA and the protected downstream 3′ UTR RNA comprise a noncoding subgenomic flaviviral RNA (sfRNA) that accumulates in the cell and performs several important functions for the virus (2, 19, 23–26), including inhibiting host antiviral responses (27, 28), affecting viral transmissibility (29–33), and disrupting the host mRNA decay program (34, 35). Within plant viruses, xrRNAs are associated with both noncoding and coding subgenomic RNAs (36).

Detailed three-dimensional crystal structures of xrRNAs from several flaviviruses provide insight into how an xrRNA resists the action of 5′–3′ exoribonucleases (1, 13, 37). Specifically, all xrRNAs fold into a compact structure containing a ring-like feature that wraps around the 5′ end of the resistant RNA element. Biochemical analyses suggest that this topology acts as a molecular brace against the surface of the exoribonuclease approaching from the 5′ side, preventing it from progressing past a defined point (38). This ring is formed through specific structural motifs, including a three-way junction, pseudoknots and other long-range tertiary interactions, non-Watson-Crick base pairs, complex base-stacking arrangements, and base triples (39). A ring-like structure is also found in the xrRNAs of some genera of the plant-infecting *Tombusviridae* and *Luteoviridae* viral families, although the structural strategies used to form and stabilize the ring are different (21, 36, 40). There may be as-yet-undiscovered ways to form a ring-like structure and thus other RNA architectures that can block exoribonucleases.

Biochemical, structural, and sequence comparison studies led us to previously group flaviviral xrRNAs into two distinct classes (38) (Fig. 1B). The class 1 xrRNAs are found in the MBFV, in some no-known-vector (NKV) flaviviruses, and in some insect-specific flaviviruses (ISFV). The class 2 xrRNAs are found in tick-borne flaviviruses (TBFV) and in some related NKV viruses. These classifications are based on the proposed secondary structure of the elements, the halt point of Xrn1 relative to these putative secondary structures, and sequence conservation patterns (see Fig. S1 in the supplemental material) (38). Classification is informed and refined by three-dimensional structures which improve sequence alignment efforts (for class 1 MBFV xrRNAs [1, 13], and for an xrRNA from Tamana bat virus [TABV] [37] [Fig. 1C and D], there is no structure of a class 2 yet).

10.1128/mBio.02352-20.1 FIG S1 Secondary structures of Zika virus (ZIKV), Tamana bat virus (TABV), and tick-borne encephalitis virus (TBEV) xrRNA. Representative xrRNA secondary structures from (A) ZIKV (1), (B) TABV (37), and (C) TBEV (38) are presented. The Zika virus xrRNA has been established as a class 1 xrRNA with the secondary structure derived from the crystal structure. The TBEV xrRNA is predicted to be a class 2 xrRNA. Red and yellow lines in panels A and B indicate the formation of the pseudoknot, with the green lines indicating an observed base triple. The black lines in panels A and B indicate an observed long-range nucleotide interaction. The yellow line in panel C represents a pseudoknot tested experimentally by site-directed mutagenesis. Download FIG S1, PDF file, 0.4 MB. Copyright © 2020 Szucs et al. 2020 Szucs et al. This content is distributed under the terms of the Creative Commons Attribution 4.0 International license.

The *Flaviviridae* family consists of four major genera: *Flavivirus*, *Pegivirus*, *Pestivirus*, and *Hepacivirus* (41–44). Within *Flaviviridae*, xrRNAs have been definitively identified only in the *Flavivirus* genus, in a single pestivirus (which might actually be a misclassified flavivirus) (3, 45, 46), and now in the phylogenetically isolated TABV (Fig. 1B) (37). If xrRNAs exist in the genomic RNA of the *Pegivirus*, *Pestivirus*, and *Hepacivirus* genera, this would indicate their presence in the three other *Flaviviridae* genera that were previously thought to be devoid of xrRNAs. Their folds may also look different, therefore potentially calling for classification adjustments as more viruses are discovered, more xrRNAs are characterized, and more detailed structural information is gained.

New questions about variation in flavivirus xrRNA structure arise from the recently solved structure of an xrRNA found in the 3′ UTR of TABV (37). While this xrRNA superficially resembles a class 1 xrRNA, it lacks the conserved sequences essential to form the required tertiary contacts observed in that class (Fig. 1C; see also Fig. S1) (38). Consistent with this, although the TABV xrRNA structure forms the ring-like fold, it uses an unexpected set of tertiary interactions compared to the MBFV xrRNA (Fig. 1C and D) (37). This divergence is different enough to suggest a new xrRNA subclass, potentially with other unidentified members, and to raise the issue of whether additional classes of xrRNAs exist among the *Flaviviridae*.

Using computational tools that take into account both structural and sequence constraints and findings that are informed by crystal structures, we exploited the burgeoning availability of genomic data from new viral species. We identified putative xrRNAs in the *Pegivirus*, *Pestivirus*, and *Hepacivirus* genera that we verified using *in vitro* functional studies and characterized by chemical probing. Analysis of sequences and secondary structures revealed fundamental similarities to the TABV xrRNA. Together, these xrRNAs comprise an xrRNA subclass similar to class 1 but bioinformatically and structurally distinct; we now assign them to subclass 1b and accordingly place those species previously classified in class 1 (MBFV) into subclass 1a. Thus, our results show that all genera (although not necessarily all species) within *Flaviviridae* contain xrRNAs that exist in at least three distinct structural classes/subclasses.

## RESULTS

### Computational identification and validation of an xrRNA subclass.

The recently solved crystal structure of an xrRNA from TABV (37) revealed a previously unknown secondary structure, which motivated reexamination of existing alignments and secondary structures of many putative xrRNAs from insect-specific flaviviruses (ISFV). Therefore, using *RALEE* v. 0.8 and a text editor (47), we manually constructed a new initial sequence alignment from 20 likely similar xrRNA sequences belonging to ISFV, taking into account the patterns revealed in the TABV structure. These sequences often occur in multiple copies in series, and thus we included all of these in our alignment (referred to as xr1, xr2, etc., in the 3′ UTR of Culex flavivirus [CxFV], Quang Binh virus [QBV], mosquito flavivirus [MOsFV], Culex theileri virus [CxThFV], Kamiti River virus [KRV], Aedes flavivirus [AeFV], and cell-fusing agent virus [CFAV]; Tables S1 and S2) (48). These ISFV xrRNA sequences were previously proposed to conform to class 1 xrRNAs or to have different secondary structures (38, 48), but we could align them more convincingly to the TABV xrRNA, using information from its crystal structure (37) (see Materials and Methods).

**FIG 2:**
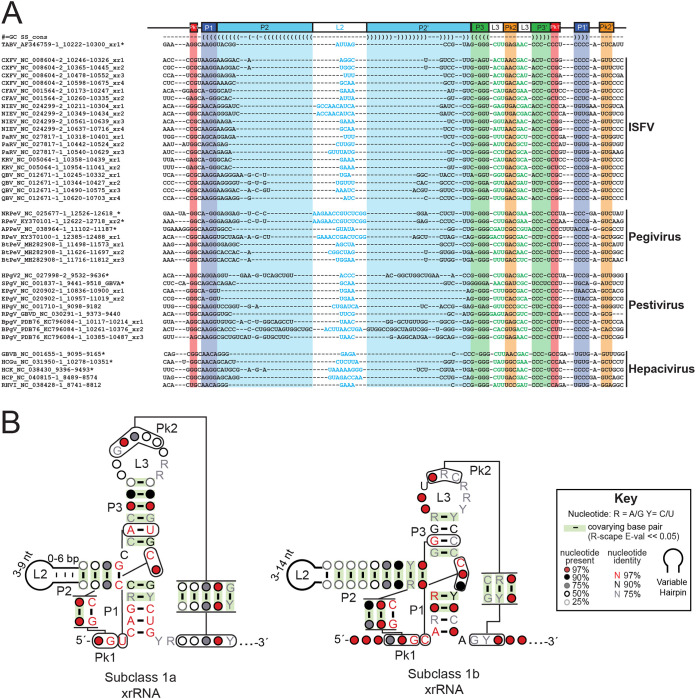
Sequence conservation of subclass 1b xrRNAs. (A) Comparative sequence alignment of select subclass 1b xrRNAs from insect-specific flavivirus (ISFV), *Pestivirus*, *Pegivirus*, and *Hepacivirus*. The TABV xrRNA xr1 sequence and the secondary structure derived from the crystal structure were used as a reference. A star (*) designates sequences used for subsequent biochemical analysis. (B) Covariance models of the subclass 1a xrRNAs (left) (32 sequences; updated from data presented in reference 39) and the subclass 1b xrRNAs (right) (87 sequences). Covarying base pairs validated by R-scape (E value < 0.05) are highlighted in green.

10.1128/mBio.02352-20.4 TABLE S1 Complete subclass 1b xrRNA sequence alignment. This sequence alignment was used to generate the subclass 1b covariance model (Fig. 2B). Constructs used for chemical probing analysis are designated by a star. Labels are formatted with the name of the virus, the accession number, the location of the xrRNA within the accession number, and the relative position of the xrRNA where several are found in series (i.e., xr1 is the 5′-most xrRNA element). Sequences ending in a plus sign (“+”) were used in the initial sequence alignment as input for *Infernal* searches. Download Table S1, PDF file, 0.05 MB. Copyright © 2020 Szucs et al. 2020 Szucs et al. This content is distributed under the terms of the Creative Commons Attribution 4.0 International license.

10.1128/mBio.02352-20.5 TABLE S2 General information about subclass 1b xrRNA sequences. Download Table S2, PDF file, 0.2 MB. Copyright © 2020 Szucs et al. 2020 Szucs et al. This content is distributed under the terms of the Creative Commons Attribution 4.0 International license.

The manually assembled alignment of ISFV was used to build a covariance model to search for similar structure patterns through all available +ssRNA viral sequences at the National Center for Biotechnology Information (NCBI; last retrieved 24 April 2020) using the program Infernal (49). Multiple iterations (see Materials and Methods) resulted in the identification of 68 putative xrRNA sequences, belonging to the following genera: *Flavivirus* (*n* = 23), *Pegivirus* (*n* = 28), *Pestivirus* (*n* = 7), and *Hepacivirus* (*n* = 10) (Fig. 2A; see also Table S1 in the supplemental material). Note that many sequences in the database did not include the 3′ UTR. Thus, more viruses than those reported here may contain putative xrRNAs that were not identified due to incompleteness of the deposited viral genome sequences.

From this expanded comparative sequence alignment, we generated a covariance model of the newly proposed xrRNAs (Fig. 2B, right). Importantly, using Infernal to search the +ssRNA database with this covariance model did not retrieve any MBFV sequence, indicating that these identified and revised xrRNAs did not fit into the class 1 xrRNA as it was defined. Thus, we divided the current class 1 xrRNAs into two subclasses: subclass 1a (previously defined as class 1 xrRNA) and subclass 1b (consisting of those identified in the current work). Differences observed between subclass 1b and subclass 1a xrRNAs included the sequence patterns of their P1 and P3 stems, the nucleotide patterns of their L3 loops, their Pk1 regions, and the presence or absence of a nucleotide between P2 and P3. These differences are analyzed and discussed in more depth below.

Analysis of the covariance model was performed with R-scape (50), which gave statistical support to the model and the proposed three stems (P1, P2, and P3). Specifically, in each stem, E values for covarying base pairs ranged from 8.89 × 10^−7^ to 1.29 × 10^−5^ (P1), 2.17 × 10^−14^ to 1.75 × 10^−3^ (P2), and 1.45 × 10^−4^ to 1.77 × 10^−9^ (P3). Stem length varies less for P1 (3 to 5 bp) and P3 (4 to 5 bp) than for P2 (up to 21 predicted base pairs) (Fig. 2B, right). This variation in stem length had been previously noticed for class 1 MBFVs, albeit with different stem length requirements (for P1, 5 bp; for P3, 4 to 8 bp; but for P2, 1 to 9 bp). The similarly conserved lengths of P1 and P3 could be explained by their participation in ring formation, while P2 extends away from the core fold, explaining its variable length (Fig. 1C) (37). The anticipated Pk1 and Pk2 pseudoknots that we manually predicted based on the TABV structure were also supported by R-scape (Pk1 E values, 4.58 × 10^−8^ to 1.20 × 10^−13^) (Fig. 2B).

All flavivirus class 1 xrRNAs contain a base triple necessary for forming the functional fold. In the MBFV, this is a U…A-U triple, while in TABV, it is replaced by an isosteric C…G-C (37). In our covariance model, the levels of sequence conservation of this C…G-C base triple were >97% for the G and >97% and >90% for the two Cs (Fig. 2B). Only two sequences identified in our alignment (CXFV_NC_008604.2 xr1 and xr4) possessed a U…A-U instead of the C…G-C, indicating that an instance of covariation was present but that the level of covariation was insufficient for support by R-scape (Fig. 2A; see also Table S1). Finally, only one of the sequences in our alignment contained any nucleotide between P2 and P3, a peculiarity that was discussed in the previously published report of the TABV xrRNA structure (37). The one sequence that did contain a nucleotide at that position came from *Rodent hepacivirus* isolate rn-1 (RHV; Table S1). This putative xrRNA has a U in that position which could not be otherwise accommodated within P2 or P3. Similarly, other *Hepacivirus* isolates corresponding to sequences such as the *Rodent hepacivirus* isolate RrMC-HCV (RtMC, Table S1) comprised sequences that departed from the subclass 1b pattern, potentially leading to the presence of non-Watson-Crick pairs at the base of P2 or within a somewhat altered three-way junction (sequences marked by “!!” in Table S1). These sequences were rare and found mainly in members of the *Hepacivirus* genera.

Another subset of sequences deviated from the covariance model at the base of P1. They comprised putative ISFV xrRNA sequences that were found in the more-downstream copies of putative xrRNAs (xr3, xr4, etc.). Among them were xr4 from *Parramatta River virus* isolate 92-B115745 (PARV_xr4; Table S1), xr3 from cell-fusing agent virus strain Galveston (CFAV_xr3; Table S1), xr1 from Mercadeo virus isolate ER-M10 (MECDV_xr1; Table S1), and xr1 from Menghai flavivirus isolate MHAedFV1 (MFV_xr1; Table S1). For example, only a 2-bp P1 could be proposed for PaRV_xr4, with no equivalent to A48 present in TABV. Further testing will be required to determine whether these sequences indeed correspond to xrRNAs and, if so, whether they possibly form a subtype within subclass 1b.

### Bioinformatically identified subclass 1b xrRNAs are exoribonuclease resistant.

**FIG 3:**
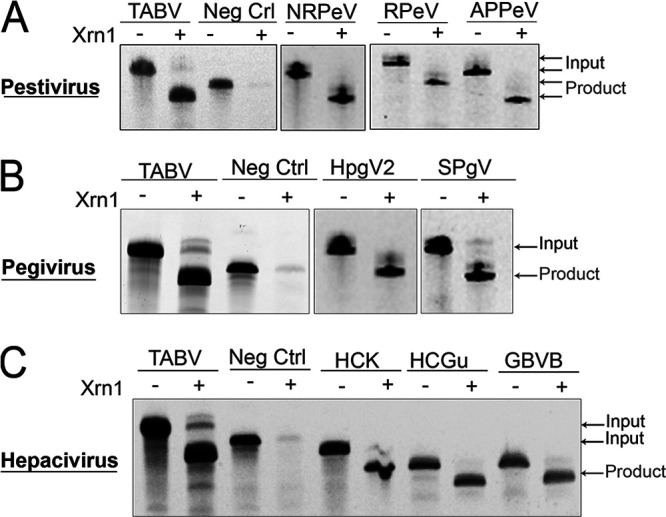
Biochemical validation of exoribonuclease resistance of subclass 1b xrRNA representatives. Each panel shows the results from denaturing 8% polyacrylamide gel electrophoresis used to analyze Xrn1 resistance experiments. (A) Representative pestivirus subclass 1b xrRNA. From left to right: NRPeV, Norway rat pestivirus (NRPeV_NC025677-1_12526-12618*); RPeV, rodent pestivirus (RPeV_KY370101-1_2622-12718_xr1*); APPeV, atypical porcine pestivirus (APPeV_NC_038964-1_11102-11187). (B) Representative pegivirus subclass 1b xrRNA. From left to right: simian pegivirus (SPgV_NC_001837-1_9441-9518_GBVA*) and human pegivirus 2 (HPgV2_NC_027998-2_9532-9636_CP*). (C) Representative hepacivirus subclass 1b xrRNA. From left to right: GB virus B (GBVB_NC_001655-1_9095-9165*), Guareza hepacivirus (HCGu_NC_031950-1_10278-10351), and hepacivirus (HCGu_NC_031950-1_10278-10351*). TABV, Tamana bat virus (positive control); Neg Ctrl, nonresistant RNA (tRNA-like structure) (52).

To test whether computationally identified subclass 1b xrRNAs were resistant to Xrn1 and therefore comprised authentic xrRNAs, we subjected representative sequences from *Pestivirus*, *Pegivirus*, and *Hepacivirus* to *in vitro* Xrn1 resistance assays (Fig. 3). Briefly, eight putative subclass 1b xrRNAs (marked by stars [*] in Fig. 2A and Table S1) were transcribed *in vitro* with a 22-nucleotide (nt) leader sequence, which allowed Xrn1 to load onto the 5′ end of the transcribed RNA. Some of the previous work from our laboratory showed that a wild-type leader sequence of an arbitrary length could generate false-negative results in Xrn1 resistance assays, likely due to RNA misfolding. Hence, for our assays of potential subclass 1b xrRNAs, the leader sequence contained the same normalization hairpin (xxxx-GAGUA-xxxx) and spacer sequences used for the chemical probing experiments described in the next section (see Materials and Methods; see also Table S3). This adjustment also contributed to consistency between resistance assays and probing experiments.

10.1128/mBio.02352-20.6 TABLE S3 *In vitro*-transcribed subclass 1b construct and primer information. Sequence information for the biochemically tested subclass 1b xrRNAs and PCR and reverse transcription primers used for cloning and reverse transcription are listed. Download Table S3, PDF file, 0.4 MB. Copyright © 2020 Szucs et al. 2020 Szucs et al. This content is distributed under the terms of the Creative Commons Attribution 4.0 International license.

RNAs were treated with RNA 5′-pyrophosphohydrolase (RppH), leaving a monophosphorylated 5′ end (51). When an RNA is resistant to Xrn1, subsequent incubation with recombinant Xrn1 results in a defined but smaller product when resolved by polyacrylamide electrophoresis, as the enzyme loads and partially degrades the RNA but then stops the process. As a positive control, we used the *in vitro*-transcribed TABV xrRNA (xr1) (37). An RNA with a tRNA-like structure that is not resistant to Xrn1, with appended normalization hairpins, was used as a negative control (52). When challenged with Xrn1, all putative xrRNA sequences identified in our computational searches were resistant, indicated by the appearance of the shorter but stable RNA product (Fig. 3). This result confirmed that even sequences with very short (3-nt) (Fig. 3C, GB virus B [GBVB]) or long (15-nt and 10-nt) P2 stems (Fig. 3B, human pegivirus 2 [HPgV2]; Fig. 3C, hepacivirus K virus [HCK]), or with the potential for a particularly long (4-nt) Pk1 (Fig. 3B, simian pegivirus [SPgV]), could form an Xrn1-resistant structure. Even the xrRNA from hepacivirus P isolate RHV-GS2015 (HCP), which displays a putative A.A pair at the base of P2, was resistant to Xrn1 (see Fig. S2 in the supplemental material). Thus, the bioinformatically identified subclass 1b xrRNAs that we tested are authentic exoribonuclease-resistant elements, and this result suggests that the untested sequences from our sequence alignment likely also represent true xrRNAs.

10.1128/mBio.02352-20.2 FIG S2 Uncropped subclass 1b *in vitro* degradation assay gels. Raw gel images were used and were cropped for Fig. 3. Red boxes indicate data shown only in the supplemental material and not in the main text. Download FIG S2, PDF file, 2.5 MB. Copyright © 2020 Szucs et al. 2020 Szucs et al. This content is distributed under the terms of the Creative Commons Attribution 4.0 International license.

### Secondary structure predictions of subclass 1b xrRNAs are compatible with chemical probing.

**FIG 4:**
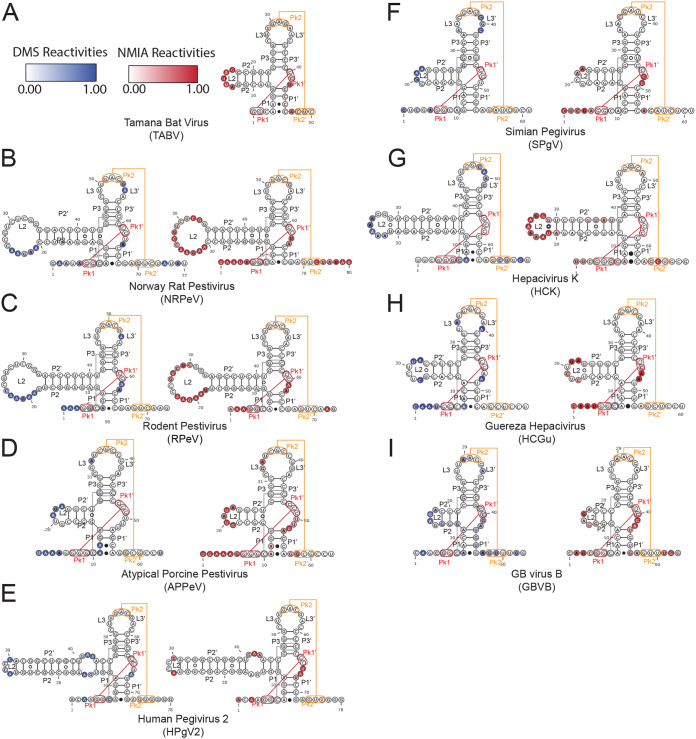
Secondary structure validation of subclass 1b through chemical probing. Subclass 1b constructs were probed with DMS and NMIA. Normalized DMS (blue) and NMIA (red) reactivities were combined with comparative sequence analysis to derive secondary structure models. Reactivities were overlaid on the secondary structure using Varna v. 3.93 (65). (A) Tamana bat virus. (B) Norway rat pestivirus. (C) Rodent pestivirus. (D) Atypical porcine pestivirus. (E) Human pegivirus 2. (F) Simian pegivirus. (G) Hepacivirus K. (H) Guereza hepacivirus. (I) GB virus B hepacivirus. Raw and normalized data are shown in Fig. S3 in the supplemental material.

Because the predictions of the secondary structures were based on sequence alignments and observed nucleotide covariation, we tested these predictions through chemical probing with dimethyl sulfate (DMS; modifies the Watson-Crick side of unpaired A and C) and N-methylisatoic anhydride (NMIA; modifies the 2′ hydroxyl group of nucleotides not involved in Watson-Crick pairs) (Fig. 4) (53, 54). We first probed (using NMIA only) the wild-type sequence of the TABV xrRNA (37), which expands on observations based on the crystal structure by providing a “fingerprint” of the chemical probing pattern for this type of fold (Fig. 4A). Most of the nucleotides were unreactive to NMIA (including in L3, which appears as “unpaired” in the secondary structure diagram, except for Pk2), supporting the hypothesis of a compact and stable fold. Highly reactive positions in the exposed L2 loop were consistent with the TABV xrRNA crystal structure (Fig. 1C). Likewise, strong reactivities at U43 and A48 also could be rationalized by analysis of the structure; U43 is flexible, and the ribose of A48 adopts a reactive C2′-endo pucker (55, 56). Although chemical probing was performed with the wild-type TABV sequence, the resulting data are consistent with the structure of the sequence variant engineered for crystallization (37). In previous studies, removal of the P4 stem did not affect Xrn1 resistance (37, 57); thus, we did not examine it as part of our analysis, but the full chemical probing construct with the P4 stem is displayed in the supplemental material (Fig. S3).

10.1128/mBio.02352-20.3 FIG S3 *Pestivirus*, *Pegivirus*, and *Hepacivirus* chemical probing data. Each of the following pages contains one of several xrRNA constructs with similar labels and notation, showing the raw data for chemical probing experiments. The eight constructs are ordered by genera as follows: *Pestivirus*, *Pegivirus*, and *Hepacivirus.* (A) Normalized DMS and NMIA reactivities mapped onto the secondary structure representation of the xrRNA, including the putative P4 region. (B and C) CE lanes and reactivity plot for DMS (B) and NMIA (C) used in the experiment. Red circles around the peaks in the reactivity plot represent a higher degree of standard deviation observed for that point. Download FIG S3, PDF file, 1.4 MB. Copyright © 2020 Szucs et al. 2020 Szucs et al. This content is distributed under the terms of the Creative Commons Attribution 4.0 International license.

We performed chemical probing on the eight *Pegivirus*, *Pestivirus*, and *Hepacivirus* xrRNAs tested for Xrn1 resistance to compare each probing fingerprint to that of TABV. The resultant patterns were consistent with the pattern from the TABV xrRNA, indicating that they adopt similar secondary structures (Fig. 4). In seven cases, the RNAs were similarly nonreactive overall, except for L2 and the nucleotide equivalent to U43 in TABV (Fig. 4B to H). GBVB showed the same pattern but was overall more reactive, likely indicative of a less stable *in vitro* fold (Fig. 4I). Several of the RNAs contained >30 nucleotide expansions in the P2 regions, and in all cases the probing indicated that they were folded as elongated stem-loops, in some cases with internal loops as seen in HPgV2 (Fig. 4B, C, E, and G). In some of these, parts of L2 may be involved in additional intramolecular interactions, as suggested by the absence of reactive positions within L2 for Norway rat pestivirus (NRPeV), rodent pestivirus (RPeV), and HPgV2 (Fig. 4B, C, and E). Probing revealed that some sequences had two reactive nucleotides (NRPeV, RPeV, HPgV2, and GBVB) or even three reactive nucleotides (atypical porcine pestivirus [APPeV]) between Pk1′ and P1′, regardless of the potential for Pk1 to contain more than three base pairs, which could be interpreted from looking only at the sequence (Fig. 4B to E and I). Only one sequence tested (HCK) had no reactive nucleotide at that position (Fig. 4G).

Intramolecular interactions involved in proper ring folding, such as at the position equivalent to the strongly reactive A48 in TABV, were reactive only in APPeV (G57), SPgV (A54), and GBVB (A47) (Fig. 4D, F, and I). Finally, the presence of reactive positions corresponding to DMS but not NMIA in L3′ (the 3′ side of L3) for NRPeV, RPeV, SPgV, HCK, Guareza hepacivirus (HCGu), and GBVB implied that the Watson-Crick face of these residues remained available in an otherwise structured region of stacked bases, which is compatible with the availability of the A34, A35, and C36 bases in the TABV crystal structure. Overall, the chemical probing data provide evidence supporting the secondary structure predictions based on comparative sequence alignment and covariation analysis.

### Structural analysis of the subclass 1b xrRNA.

**FIG 5:**
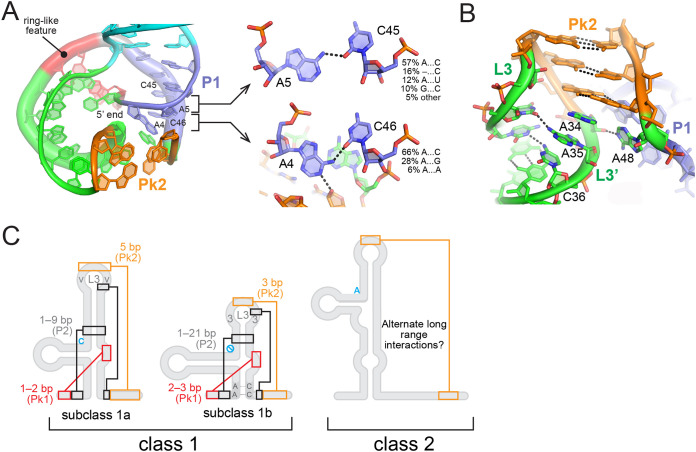
Structural analysis of subclass 1b xrRNA. (A) View of the ring-like structure in the TABV xrRNA from the 3′ side. The 5′ end is kept at the center of the ring and points away from the reader. Regions are color coded as indicated in Fig. 1C. A…C interactions in P1 are highlighted in the close-ups. (B) Close-up of L3 and Pk2. (C) Structural features for classes 1 and 2 are highlighted on secondary structure silhouettes. The blue “no” symbol (circle with diagonal line) for subclass 1b highlights the absence of a nucleotide in the junction between P2 and P3. In addition to the differences highlighted here, subclass 1a contains regions of absolutely conserved sequences that are not in subclass 1b (Fig. 2B).

On the basis of the sequence covariation observed and our structural analysis, xrRNAs from subclass 1b are more compact than those from subclass 1a, except for the P2 stem (Fig. 2B). Subclass 1b xrRNAs are distinguished from those of subclass 1a by four structural features observed in the TABV xrRNA crystal structure. The first is the presence of, generally, two non-Watson-Crick interactions at the 5′ end of the P1 stem (Fig. 5A and C). Over half of the sequences in our alignment had the two A…C interactions observed in the TABV structure, followed by two Watson-Crick pairs before the three-way junction (Fig. 2A; see also Table S1). The interactions between A4…C46 and A5…C45 seen in the TABV structure help create a sharp bend in the structure associated with a break in base stacking between Pk1 and P2 (Fig. 5A). For the remainder of the sequences, one of the two As was missing, probably the equivalent to A5, because A4 is involved in stabilizing interactions at the ring closure (Fig. 5A). In 28% of sequences, A4…C46 was replaced by A…A/G interactions. This change was accompanied by the presence of A–U or G–C instead of A5…C45, suggesting that a third Watson-Crick pair could form in P1 in that case. A common Hoogsteen/sugar edge configuration at A…G and Watson-Crick configuration at A–U or G–C would lead to a shift of the pairing partner of both A bases toward the minor groove. In any event, the length of P1 is constrained to 3 to 4 bp by the compactness of the fold, a structural feature which is supported by our comparative sequence alignment (Fig. 2A). Conversely, the subclass 1a P1 stem is highly conserved, with only two base pairs showing covariation among the six possible Watson-Crick base pairs (Fig. 2B). Also, the xrRNAs in subclass 1b lack the almost absolutely conserved sequences that are found in the subclass 1a xrRNAs, for example, in the P1 stem, making them distinct (39) (Fig. 2B).

The L3 loop comprising the Pk2 pseudoknot is the second defining feature of the subclass 1b xrRNAs. The L3 region exists in a 3-3-3 (86%), 4-3-3/3-3-4 (13%), or 4-3-4 (1%) nucleotide configuration (the number of nucleotides refers to the length of the 5′ side of L3 and of Pk2 and to that of the 3′ side of L3 or L3′) (Fig. 2A and 5C; see also Table S1). Sequence patterns of L3 could be rationalized based on the TABV structure as follows: (i) the 5′ side generally comprises three pyrimidines (90% U at the position preceding Pk2; Fig. 2B), due to the tight turn leading to Pk2; (ii) the 3-bp length of Pk2 is conserved, as A34 within L3′ immediately downstream participates in ring closure interactions (Fig. 2B and 5B); and (iii) L3′ following Pk2 generally comprises two purines (A34 and A35 in TABV) which help support stacking of Pk2 on P3, followed by a pyrimidine (C36) which could accommodate the guanine present on the 5′ side of L3 in 45% of sequences (Fig. 2; see also Table S1 in the supplemental material). Notably, the first adenines following Pk2 did not get modified by NMIA, although they were reactive to DMS (Fig. 4), which is consistent with them being conformationally constrained but with available Watson-Crick edges, as revealed by the TABV xrRNA structure (Fig. 5B).

Next, a defining structural feature of subclass 1a lacking in subclass 1b is the presence of a conserved cytosine between P2 and P3. In subclass 1a xrRNAs, this nucleotide is important for tertiary interactions that support folding and the formation of the ring-like structure around the 5′ end of the xrRNA. Studies have shown that mutating this C disrupts the ability of xrRNA to resist Xrn1 (1). In recent studies performed with TABV, it was shown that its fold cannot tolerate the addition of a nucleotide in this region (37). Because the ring of subclass 1b TABV xrRNA is stabilized by a set of long-range interactions that are different from those associated with the subclass 1a Zika virus (ZIKV) xrRNA, this results in a more compact fold that can no longer accommodate the C. Although this C is critical for subclass 1a xrRNAs, our present work shows that its absence is a shared feature among subclass 1b xrRNAs.

Finally, the covariation pattern of Pk1 is another key feature of the subclass 1b xrRNAs, although the data are less distinctive between the two subclasses. According to sequence alignment and probing data, this region accommodates either two base pairs (11% of the sequences identified) or three base pairs (89% of the sequences) (Fig. 5B and C; see also Table S1). Of the eight sequences that we probed, five revealed that the Pk1 predicted to have three base pairs (based on sequence) actually formed only two base pairs (Fig. 4B, E, F, H, and I). Together with the findings regarding the crystal structure of TABV (where Pk1 comprises two base pairs), these results suggest that the presence of two base pairs is sufficient to support a resistant fold, although we cannot exclude the possibility that Pk1 may have an additional pair on its 5′ side in some other cases, or that this pair is dynamic (Fig. 1C). The second of the two Pk1 base pairs (which is always formed) is in >97% of cases a G-C (Fig. 2B), as in subclass 1a. The first nucleotide immediately downstream of Pk1′ was strongly reactive to NMIA, except for in HCK (Fig. 4G). This feature is consistent with the flipped-out conformation of U42 in the TABV structure (Fig. 1C and D). In subclass 1a, the PK1 region never consists of more than two base pairs, but it can be up to three for subclass 1b.

## DISCUSSION

In this study, we identified and structurally characterized xrRNAs in all genera of *Flaviviridae*, one of the largest families of RNA viruses, expanding on the known distribution of these RNA structures within *Flaviviridae* (23). On the basis of our computational, structural, and biochemical data, we propose that the characteristics of these new xrRNAs require a division of the previously proposed class 1 xrRNA (38) into two distinct subclasses: subclass 1a (comprising MBFV, in particular, Murray Valley encephalitis virus [MVEV], ZIKV, West Nile virus [WNV], yellow fever virus [YFV], etc.) and subclass 1b (comprising TABV, ISFV, *Pegivirus*, *Pestivirus*, and *Hepacivirus*). Subclasses 1a and 1b deviate in (i) the sequence patterns of their P1 and P3 stems, (ii) the nucleotide patterns of the L3 loop, (iii) the Pk2 region, and (iv) whether a particular nucleotide is present between P2 and P3. In addition, the pattern of almost absolutely conserved nucleotides that is indicative of subclass 1a is not present in subclass 1b (39). Thus, all of the identified xrRNAs in *Flaviviridae* fall into three distinct groups: subclasses 1a and 1b and class 2 (which remains as described in the literature) (38).

Within known *Flaviviridae* sequences, subclasses 1a and 1b do not appear in the same viral species. In other words, no viral sequence within the phylogenetic groups characterized as having subclass 1a xrRNAs possesses a subclass 1b xrRNA and vice versa. When several xrRNAs are present in the same 3′ UTR (58), all belong to the same subclass. This observation supports the hypothesis of the evolution of these structural elements from a common ancestor (59), as opposed to horizontal gene transfer. This scenario is further supported by our observation that hepaciviruses—representing one of the most distantly related genera within *Flaviviridae—*also contain the most divergent subset of subclass 1b xrRNAs, possibly branching out further into distinct subcategories. Moreover, hepaciviruses include viruses which do not have xrRNAs, such as hepatitis C virus (60). In short, xrRNAs are evolving with the rest of the attached viral genome and not actively “hopping” across genera.

Although subclass 1b xrRNAs are resistant to Xrn1 *in vitro*, their function in the context of viral infection and pathogenesis remains unclear. A key functional role of xrRNAs from subclass 1a is that of enabling formation of sfRNAs, which have key roles for virus survival within the vector and host cells (23, 27, 61). Whether the subclass 1b xrRNAs from pestiviruses, pegiviruses, and hepaciviruses have similar functions in the generation of viral sfRNA is currently unknown. We did not identify any xrRNA sequences in the 3′ UTRs of hepatitis C virus or bovine virus diarrhea virus, both of which have been reported to not generate sfRNAs (3). Thus, while xrRNAs exist in all genera of the *Flaviviridae*, they do not exist in all species of those genera. Also, even viruses with a subclass 1b xrRNA may not generate an sfRNA. As an example, we show that GBV-C has a subclass 1b xrRNA (see Fig. S2 in the supplemental material), although previous studies indicated that it may not produce any sfRNA (3). Functions other than generating subgenomic RNAs were also expected from our previous discovery that plant virus xrRNAs could be located upstream of coding regions (36). Systematic testing of viruses for formation of sfRNAs would be a first step in pinpointing alternative functions of subclass 1b xrRNAs. Whether subclass 1b xrRNAs participate in sfRNA generation or not, evolutionarily, they maintain the necessary interactions for proper folding into a structure that can resist exoribonucleases.

In addition to motivating systematic characterization of sfRNA formation within all *Flaviviridae* genera, the findings reported here also reinforce the idea of the importance of combining bioinformatic, biochemical, and structural techniques to derive meaningful conclusions about viral structured RNAs. Bioinformatics represents a powerful tool to develop xrRNA secondary structure predictions, but this approach requires the availability of complete 3′ UTR sequences. We estimate that >40% of the available viral genome sequences for pesti-, pegi-, and hepaciviruses end at the last codon of the coding region. In addition, biochemical experiments are needed not only to validate computational predictions but also to refine the proposed models. As an example, the APPeV xrRNA was predicted to have a 6-bp Pk1, based solely on the sequence alignment and our covariance model. This possibility was refuted—in the context of Xrn1 resistance at least—by the chemical probing experiments revealing that Pk1 consisted of three base pairs (Fig. 4D). We also bioinformatically identified two putative xrRNAs in HPgV2 whose sequences overlapped (Fig. S3). The Xrn1 resistance assay enabled us to determine which sequence corresponded to the true xrRNA. Similarly, further testing and structural mapping of the putative but still ambiguous xr3–xr5 elements for some of the ISFVs (classical swine fever virus [CSFV] and PaRV) should reveal whether they are actual xrRNAs and, if so, whether they might represent another subgroup within subclass 1b. Overall, this report expands the available data revealing the widespread presence of xrRNAs in nature, and now within all *Flaviviridae* viruses, thereby further supporting the idea of the prevalence of this particular structure in the viral world.

## MATERIALS AND METHODS

### Subclass 1b bioinformatic searches and covariance model analysis.

An initial subclass 1b alignment was created starting from a total of 20 sequences of insect-specific flaviviruses (sequences with a “+” symbol; see Table S1 in the supplemental material) that were manually aligned with a combination of *RALEE* v. 0.8 (47) and a text editor, using the TABV secondary structure as a reference. Using *Infernal* v. 1.1.3 (49) with default parameters, we searched a database of reference viral genomes consisting of all currently available +ssRNA sequences downloaded from the National Center for Biotechnology Information (NCBI; last retrieved on 24 April 2020). Hits from the Infernal searches were manually added to the comparative sequence alignment when they met all of the following criteria: *Infernal* E value of <0.05, >15% nucleotide variation within each sequence, presence of Pk1 and Pk2, and location within the 3′ UTR. Sequences with higher E values were also inspected and added to the list if they met the last three requirements. For the final proposed covariance model of 87 xrRNA sequences, we performed statistical validation using *R-scape* v.1.5.3 (50) and rendering with *R2R* v.1.0.5 (62).

### Plasmid construction.

Each xrRNA construct (Table S3) was designed as a double-stranded DNA gBlock (IDT) and was subsequently cloned into the EcoRI and BamHI sites of pUC-19. Cloned plasmids were amplified in competent Escherichia coli DH5α cells and purified via the use of a Qiagen miniprep kit (Qiagen). The recovered plasmid stocks were verified through sequencing (Eton Bioscience).

### *In vitro* RNA transcription.

Template DNA was amplified by PCR using custom DNA primers (Table S3) and recombinant Phusion Hot Start polymerase (New England Biolabs). *In vitro* transcription was carried out in a volume of 2.5 ml comprising 1.0 ml of PCR as the template. The transcription reaction mixture contained ∼0.2 M template DNA, a 6 mM concentration of each rNTP (ribonucleoside triphosphate), 60 mM MgCl_2_, 30 mM Tris (pH 8.0), 10 mM dithiothreitol (DTT), 0.1% spermidine, 0.1% Triton X-100, T7 RNA polymerase, and 2 μl RNasin RNase inhibitor (Promega). The transcription reaction mixture was incubated overnight at 37°C. The RNA was precipitated with 4 volumes of ice-cold 100% ethanol, incubating overnight at –20°C. Precipitated RNA was gel purified via the use of 7 M urea–8% denaturing polyacrylamide gel electrophoresis (dPAGE)–1× Tris-borate-EDTA (TBE). The RNA was visualized by UV light, excised from the gel, and eluted from the gel by the crush and soak method overnight at 4°C, using ∼50 ml of diethylpyrocarbonate (DEPC)-treated Milli-Q (Millipore) filtered water. Amicon Ultra spin concentrators (Millipore-Sigma) (30,000 molecular weight cutoff [MWCO]) were used to concentrate the eluted RNA to 2.5 mg/ml, and the reaction mixture was then stored at –20°C in DEPC-treated H_2_O until use.

### *In vitro* Xrn1 resistance assay.

A total of 5 μg RNA was incubated at 90°C for 3 min and then at 20°C for 5 min in 40 μl of refolding buffer (100 mM NaCl, 50 mM Tris [pH 7.5], 10 mM MgCl_2_, and 1 mM DTT). Next, 3 μl of recombinant RppH (0.5 μg/μl stock) was added to the mixture, which was then aliquoted into two volumes of 20 μl. A 1.5-μl volume of recombinant Xrn1 (0.8 μg/μl stock) was added to one of the aliquots, and the reaction mixture was incubated in a thermocycler at 37°C for 2 h. A 10-μl volume of RNA from each reaction (with or without Xrn1) was resolved by the use of a 7 M urea 8% dPAGE gel and visualized with ethidium bromide.

### Chemical probing.

The method used for chemical probing was adapted from a previously published method (63). RNA (1.2 pmol) was refolded in 13 μl of folding buffer (77 mM Na-HEPES [pH 8.0]) mixed with 4.8 nM 6-carboxyfluorescein (FAM) amidite-labeled reverse transcription (RT) primer (Table S3) at 95°C for 3 min, followed by 10 min of slow cooling to room temperature. A 2-μl volume of 100 mM MgCl_2_ was added to the mixture at room temperature. Final concentrations of the ingredients of the folding buffer were as follows: 6.67 mM Na-HEPES (pH 8.0), 4.1 nM FAM-labeled RT primers, and 13.3 mM MgCl_2_. This reaction mixture was incubated at 37°C for 15 min. The RNA mixture was centrifuged at maximum speed (30,130 × *g*) in a tabletop centrifuge, and the full 15-μl volume was added to 1 well of the 96-well plate where the remainder of the chemical probing experiments occurred. When setting up this experiment, enough folded RNA was made to fill the contents of a 96-well plate.

Fresh stocks of 0.5% dimethyl sulfate (DMS) (Sigma-Aldrich, catalog no. D186309) and 12 mg/ml N-methylisatoic anhydride (NMIA) (Invitrogen, catalog no. M25) were made immediately before probing was performed. Chemical modifier stocks were prepared as follows. A 10-μl volume of DMS was first added to 90 μl of 100% ethanol, which was then added to 900 μl of H_2_O. This 1% DMS solution was then diluted to 0.5% with DEPC-treated Milli-Q H_2_O. NMIA solution was prepared by dissolving 12 mg of NMIA in dimethyl sulfoxide (DMSO) (Acros Organics, catalog no. 167851000). A stock of DMSO alone was used as the no-modification control. For each condition (DMS, NMIA, or DMSO), 5 μl of the appropriate stock was added to a well containing the 15 μl of folded RNA, followed by incubation of the reaction mixture at room temperature for 30 min. Specific chemical modifier quenching solutions were prepared which consisted of 1.5 μl of cleaned oligo(dT)25 magnetic beads (Invitrogen), 3 μl of 5 M NaCl, 0.25 μl of DEPC-treated Milli-Q H_2_O, and 5 μl of the corresponding quenching solution. Fresh 2-mercaptoethanol (Sigma, catalog no. M3148) was used to quench the DMS reaction and 0.5 M 2-(N-morpholino)ethanesulfonic acid sodium salt (Na-MES) (J.T. Baker, catalog no. 4813-01) to quench the NMIA reaction. The appropriate quenching solution was added to each well and incubated at room temperature for 10 min. The chemically modified RNAs bound to the beads were washed three times with 100 μl of 70% ethyl alcohol (EtOH) on a magnetic rack. The washed beads were air dried for ∼15 min and then resuspended in 2.5 μl of 2 M betaine (Sigma, catalog no. B3501).

### Quantitation of chemical probing by reverse transcription.

The RNA was reverse transcribed on beads with 2.5 μl of a reverse transcription mixture comprised of 1.0 μl of 5× first-strand buffer (Thermo Fisher), 0.25 μl of 0.1 M DTT, 0.4 μl of a 10 mM equimolar mixture of deoxynucleoside triphosphates (dNTPs), 0.75 μl of DEPC-treated H_2_O, and 0.1 μl of SuperScript III reverse transcriptase (Thermo Fisher). The mixture was incubated in a 52°C water bath for 50 min. Upon completion of the incubation, 5 μl of 0.4 M NaOH was added to each well and incubated at 65°C for 20 min to hydrolyze the RNA. The samples were then cooled in an ice bath for 2 min, and each reaction was neutralized with 5 μl of an acid quench mixture (1.4 M NaCl, 0.6 N HCl, and 1.3 M NaOAc). The supernatant was aspirated from each well, and the cDNA bound to the beads was washed three times with 100 μl of 70% EtOH and allowed to air dry for ∼15 min. The cDNA was then eluted from the beads in 11 μl of a ROX-formamide mixture (2.75 μl of ROX-500 ladder; Applied Biosystems) with 1.2 ml of HiDi-formamide (Applied Biosystems), incubating at room temperature for 15 min. The 11 μl cDNA mixture was transferred to an optical plate and analyzed with an ABI 3500 Genetic Analyzer capillary electrophoresis (CE) machine.

### Preparation of the Sanger sequence ladder.

Alongside the reverse transcription for the chemically probed RNAs, Sanger sequencing ladders were constructed through reverse transcription of unmodified xrRNA in the presence of dideoxynucleoside triphosphates (ddNTPs). A 2.5-μl volume of an RNA mixture consisting of 0.2 μl of 0.25 μM FAM primer, 1.5 μl of oligo(dT) beads, 0.5 μl of 2.4 μM RNA, and 0.25 μl of 5 M betaine was added to 2.5 μl of a ladder reverse transcription mixture consisting of 1.0 μl of 5× first-strand buffer, 0.25 μl of 0.1 M DTT, 0.4 μl of 1.0 mM dNTP, 0.4 μl of a 1.0 mM concentration of the appropriate ddNTP, 0.1 μl of SuperScript III reverse transcriptase, and 0.25 μl of 5 M betaine. The outlined reverse transcription protocol was followed as described earlier.

### Analysis of chemical probing.

The HiTRACE MATLAB suite (https://ribokit.github.io/HiTRACE/) with MATLAB (v8.3.0.532) was used to analyze the chemical probing data as described previously (64). CE traces were first aligned manually and verified using the Sanger sequencing ladder in HiTRACE. Each run was corrected for signal attenuation, subjected to background subtraction with values from the DMSO channels, and then normalized via the 5′ and 3′ hairpin loops (GAGUA) flanking the sequence of interest in the construct. The nucleotide reactivities for each modifier were calculated, and the reactivities were mapped back to the secondary structure of the xrRNA using Varna v. 3.93 (65).
