# Supplementary material for: A New Subclass of Exoribonuclease-Resistant RNA Found in Multiple Genera of Flaviviridae
Source: mBio. 2020 Sep 29;11(5):e02352-20. doi: 10.1128/mBio.02352-20 (PMC7527734; doi:10.1128/mBio.02352-20)
Supplement: TABLE S1 [file mBio.02352-20-st001.pdf]

# Table S1: Complete subclass1b sequence alignment

```
TABV + ISFV
TABV_AF346759-1_10222-10300_xr1*+            GAA----AGG-C-AAGG-UACGG--------------------------AUUAG-----------------------CCG--UA-G-GGG---CUU-GAG-AAC----CCC-C-CCU------CC-CC-A-CUC-AUUUUAU--------------------------UUCCUC-----------------UAU-------------------------G-AGG-A-A-GGU
TABV_AF346759-1_10305-10380_xr2              UUU----GGG-C-AAGG-UGCAG--------------------------GUUAG-----------------------CUG--CA-G-GGG---CUU-GAA-AAA----CCC-C-CCC------CC-CC-A-UUC-AAGACUU--------------------------UUAG-------------------UGCA---------------------------UU-A-G-UUU
CXFV_NC_008604-2_10246-10326_xr1+            ACC----CCG-U-AAGG-AAGGAC--A------------------------AGGC------------------U---GUCC--UU-G-AGU---ACU-AAC-GAC----ACU-C-CGG------CC-CC-A-GUU-CCCAG----------------------------AGCCAG-----------------GGUU------------------------U-UAG-C-U-CCA
CXFV_NC_008604-2_10365-10445_xr2+            CGC----GCG-C-AAGG-AAGGAC--A------------------------UGGC------------------U---GUCC--UU-G-GGU---ACG-AAC-GAC----ACC-C-CGC------CC-CC-A-GUU-CUCAA----------------------------GGUUA------------------GAGUUA------------------------UAA-C-C-UCA
CXFV_NC_008604-2_10478-10552_xr3+            CCA----UCG-C-AAGG-GAGGA----------------------------UUU-----------------------UCC--UC-G-GGU---ACU-GAC-CAU----ACC-C-CGA------CC-CC-A-GUC-CGAUA----------------------------GGUCAU-----------------GGA-------------------------A-UGA-C-C-CCA
CXFV_NC_008604-2_10598-10675_xr4+            CUC----CCG-U-AAGG-AAAGC---------------------------GCAA-----------------------GCU--UU-G-AGC---AUU-GAC-AAC----GCU-C-CGG------CC-CC-A-GUC-CCCCA----------------------------GGUUAU-----------------GGGAGA----------------------A-UAA-C-C-CCA
CTFV_HE574574-1_10178-10264_xr2              CUA----GCG-C-AAGG-AAGGAAA-G-U-C---------------------GCA----------------GAC---UACC--UU-G-GGU---GUU-GAC-GAC----ACU-C-CGC------CC-CC-A-GUC-ACCUUG---------------------------GCCG-------------------AAGGUUAAA----------------------CG-G-C-AUC
CTFV_HE574574-1_10280-10359_xr3              CCA-----CG-C-AAGG-GGAGGC----------------------AUCAUUUC----------------------GCCU--CC-G-GGU---GCU-GAC-GAC----ACC-C-CG-------CC-CC-A-GUC-CCUA-----------------------------GGUCA------------------AGUGAA------------------------UGA-C-C-CCG
CTFV_HE574574-1_10406-10490_xr4+             CAC----CCG-C-AAGG-GGAGGG--U-U---------------------UUCU-----------------AA---CUCU--CC-G-GGU---GUU-GAC-GAC----ACC-C-CGG------CC-CC-A-GUC-CCCAA----------------------------GGUCUU-----------------GGGAAA----------------------A-AGA-C-C-CCG
QBV_NC_012671-1_10245-10332_xr1+             UCA----GCG-C-AAGG-AAGGGAA-G-C-U---------------------UGA----------------GGC---UACC--UU-A-GGU---GGU-GAC-GAC----ACC-U-CGC------CC-CC-A-GUC-UCCCG----------------------------GGUUG------------------GGGAUAAUA---------------------CAA-C-C-UCG
QBV_NC_012671-1_10344-10427_xr2+             CCC----CCG-C-AAGG-AGGGGU--G-U-G-------------------UGUUU----------------CAC---GCCC--CU-G-GGA---GUU-AAC-GAU----UCU-C-CGG------CC-CC-A-GUU-CCUA-----------------------------GGUCC------------------AGGGA-------------------------GGG-C-C-CCG
QBV_NC_012671-1_10490-10575_xr3+             CCC----GCG-C-AAGG-AAGGAC--A-C-GU------------------AAAUC---------------ACGU---GUUC--UU-G-GGA---GUU-GAC-GAC----UCU-C-CGC------CC-CC-A-GUC-CCCA-----------------------------GGUCA------------------GGGUA-------------------------UGA-C-U-CCG
QBV_NC_012671-1_10620-10703_xr4+             CCA----CCG-C-AAGG-GAGAGG--G-------------------------AUC------------------C---CCUC--UC-G-GGU---UUG-GAC-GAC----ACC-C-CGG------CC-CC-A-GUC-CCCCA----------------------------GGUCAU-----------------GGGAAAA---------------------ACUGA-C-C-CCG
YNCxFV_NC_021069-1_10244-10331_xr1+          UCA----GCG-C-AAGG-AAGGAAA-G-C---------------------UGGAC-----------------GC---UACC--UU-A-GGU---GGU-GAC-GAC----ACC-U-CGC------CC-CC-A-GUC-UCCCA----------------------------GGUUG------------------GGGAUCGUA---------------------CAA-C-U-UCG
YNCxFV_NC_021069-1_10343-10426_xr2+          CCC----CCG-C-AAGG-AGGGAC--G-U-G-------------------UGCAU----------------CAC---GUUU--CU-G-GGA---GUU-AAC-GGC----UCU-C-CGG------CC-CC-A-GUU-CCUA-----------------------------GGUC-------------------CAGGUA------------------------GGA-U-C-CCG
YNCxFV_NC_021069-1_10489-10576_xr3+          CCC----GCG-C-AAGG-AAGGAU--G-C-G-----------------AUGAAAA----------------CGC-UUGUCC--UU-G-GGA---GUU-GAC-GAC----UCU-C-CGC------CC-CC-A-GUC-CCCA-----------------------------GGUCA------------------GGGUA-------------------------UGA-C-C-CCG
YNCxFV_NC_021069-1_10621-10704_xr4+          CCA----CCG-C-AAGG-GAGAGG--G-------------------------AUU------------------C---CCUC--UC-G-GGU---GUG-GAC-GAC----ACC-C-CGG------CC-CC-A-GUC-CCCUA----------------------------GGUCA------------------UGGGAAAAAC--------------------UGA-C-C-CCG
CTFVtur3_KX652378-1_10323-10409_xr1          CUA----GCG-C-AAGG-AAGGAAA-G-U-C---------------------GCA----------------GAC---UACC--UU-G-GGU---GUU-GAC-GAC----ACU-C-CGC------CC-CC-A-GUC-ACCUUG---------------------------GCCA-------------------AAGGUUAAA----------------------UG-G-C-AUC
CTFVtur3_KX652378-1_10424-10505_xr2          CCC----ACG-C-AAGG-GGAGGC--A----------------------UCAUAU------------------U---GCCU--CC-G-GGU---GCU-GAC-GAC----ACC-C-CGU------CC-CC-A-GUC-CCUA-----------------------------GGUCA------------------AGUGAA------------------------UGA-C-C-CCG
CTFVtur3_KX652378-1_10552-10636_xr3          CAC----CCG-C-AAGG-GGGGAG--U-U---------------------UUCU-----------------AA---CUCC--CC-G-GGU---GUU-GAC-GAC----ACC-C-CGG------CC-CC-A-GUC-CCCAA----------------------------GGUCUU-----------------GGGAAA----------------------A-AGA-C-C-CCG
CFAV_NC_001564-2_10173-10247_xr1+            ACA--G-GAG-C-A-GG-GCAU----------------------------GAAA------------------------AU--GU-C-GGG---CAU-GAC-GAA----CCC-G-CUC-C----CC-CG-A-GUC-CCCUGGC--------------------------AACA-------------------GGGUG--------------------------UG-U-U-CCC
CFAV_NC_001564-2_10260-10335_xr2+            UUC----GAG-C-A-GG-GCAC----------------------------AUUA------------------------GU--GU-C-GGG---CGU-GAC-GCA----CCC-G-CUC-C----CC-UC-A-GUC-CCCU-----------------------------GUGC----------------AACAGGGAGG------------------------GC-A-C-UUG
AEFV_NC_012932-1_10496-10574_xr1+            UCA----GAG-C-A-GG-GCAC----------------------------AAUA------------------------GU--GU-C-GGG---CCU-GAC-GAC----CCC-G-CUC-C----CC-CG-A-GUC-GCCCAAC--------------------------GGAGUU-----------------UGGCUC----------------------A-ACU-C-U-AAA
AEFV_NC_012932-1_10634-10712_xr2+            GCA----GCG-C-A-GG-GCAU----------------------------GAAA------------------------AU--GU-C-GGG---CCU-GAC-GAA----CCC-G-CGC-A----CC-CG-A-GUC-CCCCA----------------------------GUUGGG-----------------GAAGGGAU--------------------C-CUU-G-C-AUC
AEFV_NC_012932-1_10933-11012_xr3             GCG----GAG-C-AACA-GGGA----------------------------GAAA------------------------UC--CC-G-GGG---AAU-UGC-GAA----CCC-C-CUC-C----GA-AA-U-GUG-AAAAAUU--------------------------AUGGG------------------GAAAAGUA----------------------CCC-A-U-CUA
AEFV_KJ741266-1_10497-10572_xr1              UCA----GAG-C-A-GG-GCAC----------------------------AACA------------------------GU--GU-C-GGG---CCU-GAC-GAC----CCC-G-CUC-C----CC-CG-A-GUC-ACCAACU--------------------------GGAGUU-----------------UGGCUC----------------------A-ACU-C-C-AAA
AnFV_NC_031327-1_10141-10226_xr1             GGA----AGG-C-AACA-CACCAG--------------------------UAAU----------------------CUGG--UG-G-GGU--GAGU-UGC-GAC----ACC-C-CCU------UG-UG-A-GCA-ACCGACCU-------------------------AGCCGGU----------------CUAUUG----------------------A-CCGGC-U-UGC
NIEV_NC_024299-2_10211-10304_xr1             ACA----GGG-C-AACA-GGGAUC--------------------GCCAACAUCA----------------------GAUC--CC-G-GGU---GAG-UGA-CGAC---ACC-C-CCC-A----UG-UG-AAUCG-UCAA-----------------------------CUUA----------------GGAACACAUUCAAA--------------------UAGA-G-GAG
NIEV_NC_024299-2_10349-10434_xr2             ACA----GGG-C-AACA-GGGAUC--------------------ACCAACAUCA----------------------GAUC--CC-G-GGU---GAG-UGA-CGAC---ACC-C-CCC-A----UG-UG-AAUCG-UCGA-----------------------------UAC--------------------AAAAACAC------------------------GAU-A-GGG
NIEV_NC_024299-2_10561-10639_xr3             ACA----CCG-C-AAGG-AAGA----------------------------GAAA------------------------UC--UU-G-GGU---GGU-AAC-AAC----ACC-C-CGG------CC-CC-A-GUU-CUCGC----------------------------GUGCCA-----------------CGAGUCAU--------------------U-GGC-A-C-AAA
NIEV_NC_024299-2_10637-10716_xr4             AAA----CCG-C-AAGG-AAGGA---------------------------GCAA-----------------------UCC--UU-G-GGU---AUU-AAC-GAC----ACC-C-CGG------CC-CC-A-GUU-CCGAA----------------------------GUCAAGG----------------GGAC-----------------------CC-UUG-A-C-CCA
OCFV_NC_034242-1_10279-10360_xr1             CAU-----UG-C-AGCA-GAGA----------------------------UUUA------------------------UC--UC-G-GGG--GAGU-UAC-GCC----CCU-C-CA--U----UG-CC-A-GUA-GAGUU----------------------------UGCAUGU----------------CUCUAUAA-------------------AC-AUGAC-G-UUC
PaRV_NC_027817-1_10318-10401_xr1             UGG----CGG-C-AGCA-GGAGA----------------------------UUU-----------------------UCU--CC-G-GGG---UUU-CAC-GCU----CCC-C-CCG-A----UG-CC-A-GUG-GUCACA---------------------------GCCCAAGUC--------------AGA----------------------GACU-UGGAG-U-UGU
PaRV_NC_027817-1_10442-10524_xr2             AAU--A-UGG-C-AGCA-GAG----------------------------CUUGU-------------------------C--UC-G-GGG---AUU-CAC-GCU----CCC-C-CCA-U----UG-UG-A-GUG-UGUCGAA--------------------------CUGG------------------UUUCGAAGGACGU-------------------CU-A-G-AAC
PaRV_NC_027817-1_10540-10629_xr3             AUC----CGG-C-AACA-GAGA-------------------------GUUUAUG------------------------UC--UC-G-GGG---CCU-CAC-GCA----CCC-C-CCG-U----UG-UG-A-GUG-AAGUC----------------------------CUUUCUGGCCAU-----------UUA-------------------GUGGUCA-GGA-A-G-GGU
KRV_NC_005064-1_10358-10439_xr1+             UUA----GAG-C-A-GG-GCAC----------------------------GAAA------------------------GU--GU-C-GGG---CAU-GAC-GCA----CCC-G-CUC-C----CC-CG-A-GUC-CCCUGAAAAUA----------------------GGGUG------------------GGCAAUG-----------------------CAC-U-C-CUG
KRV_NC_005064-1_10954-11041_xr2+             UUU----GAG-C-A-GG-GCAC----------------------------GAAA------------------------GU--GU-C-GGG---CCU-GAC-GCA----CCC-G-CUC-C----CC-CG-A-GUC-CCCUGGAAACAG--------------------GGUGGG-----------------CCUCGAAAAA------------------U-CCA-C-C-GUA
XFV_NC_034017-1_10365-10443_xr1              UGG----GGG-C-AGCC-GGGG----------------------------GAAA------------------------CC--CU-G-GGG---CUU-GGC-GAC----CUC-C-CCC------CA-CA-A-GCC-AUCA-----------------------------UGCG-------------------AAAUUAAGGCAGC------------------CG-C-G-AGG
MFV_NC_034204-1_10386-10482_xr1!!            UGG----GGG-C-AGCC-GGGAGU---------------------------CAA----------------------ACUC--CC-G-GGG---CCU-GGC-GAC----CCC-C-CCC------UU-CC---GCC-UC-CAAAAAUUAAGGCA----------------GCCCCGAGGG-------------AGCUC-------------------UCCUC-GGU-G-U-GAA
ALFv_KJ412989_202-279_xr1                    CUC----CCG-U-AAGG-AAAGC---------------------------GCAA-----------------------GCU--UU-G-AGC---AUU-GAC-AAC----GCU-C-CGG------CC-CC-A-GUCCCCCA-----------------------------GGUUAU-----------------GGAGGA----------------------A-UAA-C-C-CC-
PaRV_NC_027817-1_10757-10848_xr4_!!          GCA----GGG-C-AACA-AAGUUC--------------------------UAAC----------------------GAAC--UA-G-GGU---GAG-UAG-CGUC---ACC-C-CCC------GG-UU-G-UGA-AAAC-----------------------------GAUUGCGACU-------------AGAACUAA----------------AGUCGAGAG-U-C-UCC
MECDV_NC_027819-1_10811-10887_xr1_!!         GCA----GGG-C-AACA-GGAA----------------------------GAAA------------------------UU--CC-G-GGU--GAUU-AGC-CAC----ACC-C-CCC-G----AA-ACGU-GAU-UUAUA----------------------------UGAU-------------------GACAAGA-----------------------A-U-C-A-GAU
CFAV_NC_001564-2_10555-10632_xr3_!!          ACG--G--GG-C-AACA-GGGA----------------------------GAAA------------------------UC--CC-G-GGG----UA-GCG-AAC----CUC-C-UC--G----UU-A--A-UGU-GAAAAAGU-------------------------AUGGG------------------GAAAGAA-----------------------CUC-A-U-CUU

Pestivirus
NRPeV_NC_025677-1_12526-12618*               GAA-UA--GG-C-A-GG-GAGGAG--U-C-C------------AAGAACCGUCUCGG-------------GGA---CUCU--UU-G-GGG---CUU-GAC-GAA----CCC-C-CC--UA---CCCCGGA-GUC-UAUAUUCAGUGGCUGGAACCG----------------------------------------------------------------------------
RPeV_KY370101-1_12622-12718_xr1*             AAA-----GG-C-A-GG-GAGAGG--C-U-U------------AAGAACCGUCUCGG-------------GAG---CCUC--UU-G-GGG---CUU-GAC-GAA----CCC-C-CC--AAA--CC-CG-A-GUC-AAG------------------------------UCCUU----------------CAACAGUACCGUUUC-----------------GAG-G-A-CAA
APPeV_NC_038964-1_11102-11187*               UGAAAG-GGG-C-AAGU-GGCC---------------------------GUAUA------------------------GG--CU-G-GGG--CGAU-CGC-CGUA---CCC-C-CCC-UUU--AC-CA-G-GCG-CCUCA----------------------------ACCCCAUG---------------UACCA-----------------------UGGGG-U-U-G--
RPeV_KY370100-1_12385-12488_xr1              ACA----CGG-C-AAGG-UGCUAGA-A-G-CU------------GAAACCGACUCGG------------AGCU---CUAG--CA-G-GGG--GACU-GGC-GAC----CCU-C-CCG------CC-CC-A-GCU-GGCCUC---------------------------UGGCA----------------GAAACGACUCG---------------------UGC-C-A-UUG
BtPeV_MH282908-1_11498-11573_xr1             AAG----AGG-C-AAGG-GGAGGC-------------------------AGCUA----------------------GCCU--CC-G-GGG---CCU-GAC-GAC----CCC-C-CUU------CC-CC-A-GUC-AUUGAA---------------------------GGC--------------------AAGGG---------------------------G-C-U-GCA
BtPeV_MH282908-1_11626-11697_xr2             AAA----GGG-C-AAGG-CACCAC--A---------------------CGGCUAG------------------U---GUGG--UG-A-GGG---AUU-GAC-AAC----CCC-U-CCU------CC-CC-A-GUC-GGC------------------------------AUG--------------------AAC-----------------------------U-U-G----
BtPeV_MH282908-1_11716-11812_xr3             AAA----GGG-C-AAGG-CACCAC--A-----------------------UGGAA------------------U---GUGG--UG-A-GGG---UUU-GAC-CUC----CCC-U-CCC------CC-CC-A-GUC-AACCA----------------------------UAC----------------AUAAAACUUGAAAAACACAUAUUG------------G-U-A-CUG

Pegivirus
SPgV_NC_001837-1_9441-9518_GBVA*             CUC-CA--GG-C-AGCAGCAGAC---------------------------GCAA-----------------------GUC--UG-GGGGA---AAC-GAU-CGC----UCCUC-CC--U---CUG-CA-G-AUC-UCUA-----------------------------GCUC-------------------GGAUA--------------------------GA-G-C-GGG
HPgV2_NC_027998-2_9532-9636*                 ACA----AGG-C-AGGA-GGU---GAA-G-UCAGCUGU------------ACCC--------AC-GGCUGGCUGAA---A--CC-G-GGG---CUU-GAC-GAC----CCC-C-CCU-A----UC-CG-A-GUU-GGGCAAGGUAACAUCACGGGUGUGACGACCCCG----------------------------------------------------------------
EPgV_NC_020902-1_10836-10900_xr1             CAA----GGG-C-A-GG-U-------------------------------UGAA-----------------------------A-G-GGG---CUU-GGC-GAC----CCC-C-CCC------UA-ACCA-GCC-ACGC-----------------------------GCAC-------------------UGUGC--------------------------GU-G-C-GCG
EPgV_NC_020902-1_10957-11019_xr2             CCG----GGG-C-A-GG-U-------------------------------UGAA-----------------------------A-G-GGG---UUU-CAC-GCA----CCC-C-CCC------CU-ACCA-GUG-CCGU-----------------------------CGUU-------------------GGU----------------------------AA-C-G-CGG
EPgV_MF438044-1_10714-10779_xr1              CAA----GGG-C-A-GG-U-------------------------------UGAA-----------------------------A-G-GGG---CUU-GGC-GAC----CCC-C-CCC------UA-ACUA-GCC-ACGC-----------------------------GCAC-------------------UGAUGU-------------------------GC-G-C-GGC
EPgV_MF438044-1_10834-10896_xr2              CCG----GGG-C-A-GG-U-------------------------------UGAA-----------------------------A-G-GGG---CAU-CAC-GCA----CCC-C-CCC------CU-ACCA-GUG-CCGU-----------------------------CGUU-------------------GGU----------------------------AA-C-G-CGG
RPgV_NC_021154-1_11072-11138_xr2             ACC----GGG-C-AAGG-GC------------------------------UCAC----------------------------GC-G-GAG---UGU-GAC-AAG----CUC-C-CCC------CC-CC-A-GUC-CAUG-----------------------------GCCG-------------------UGGAU--------------------------CG-G-C-UCG
HPgV_AF070476-1_9015-9094                    ACG-----GG-C-AAGG-AGC-----------------------------GCAA-------------------------G--CU-G-GGG---CUU-UCC-GAC----CCC-C-CC-------CC-CC-A-GGA-CGAUUCCCC------------------------GCUUGGU----------------AAAAAGG--------------------GC-CAG-G-C-CAU
SPgVkob_KF234530-1_9178-9267                 AUG----UGG-C-AAGG-GGCCUG-------------------------CUAACA---------------------CAGG--CC-G-GGG---CUU-CCU-GAC----CCC-C-CCA------CC-CC-A-AGG-CUGUUCCCC------------------------GCUCGGU----------------AAAAAGG--------------------GC-CGG-G-C-CAU
HPgV_KT166442-1_9100-9186                    AUG----AGG-C-AAGG-AGCCUG-------------------------CUAACA---------------------CAGG--CU-G-GGG---CUU-CCU-GAC----CCC-C-CCU------CC-CC-A-AGG-CGGUUCCCC------------------------GCUCGGU----------------AAAAAGG--------------------GC-CGG-G-C-CUA
SPgVkrc_NC_024377_9230-9314                  GUG----AGG-C-AAGG-AGACAU--U-C----------------------CAG-----------------GA---AUGU--CU-G-GGG---CUU-UCC-GAC----CCC-C-CCU------CC-CC-A-GGA-CGGU-----------------------------UCC-----------------CCGCUGAGUAAAAA---------------------G-G-G-CUC
SPgVkrtg_KF234529-1_9142-9233                AAU----GUGGC-AAGG-GGCCUG--U-C----------------------CAA-----------------GA---CAGG--CC-G-GGG---CUU-UCC-GAC----CCC-C-CAC------CC-CC-A-GGA-CGGUUCCCC------------------------GCUCGGU----------------AAAAAGG--------------------GC-CGG-G-C-UAU
HPgV_AB008335-1_9096-9189                    UUG----CGG-C-AAGG-UCGGCC--G-A-------------------CUGAUCA----------------UC---GGCU--GA-G-GAG---GUU-CCC-GCC----CUC-C-CCG------CC-CC-A-GGG-GUCUCCCC-------------------------GCUGGGU----------------AAAAAGG--------------------GC-CCG-G-C-CUU
HPgV_NC_001710-1_9098-9182                   UUG----CGG-C-AAGG-UCCGGU--G-A-------------------CUGAUCA----------------UC---ACCG--GA-G-GAG---GUU-CCC-GCC----CUC-C-CCG------CC-CC-A-GGG-GUCUCCCC-------------------------GCUGGGU----------------AAAAAGG--------------------GC-CCG-G-C-CUU
HPgV_AB018667-1_8931-9024                    UUG----CGG-C-AAGG-UCGGGC--G-A-------------------CUGAUCA----------------UC---GCCU--GA-G-GAG---GUU-CCC-GCC----CUC-C-CCG------CC-CC-A-GGG-GUCUCCCC-------------------------GCUGGGU----------------AAAAAGG--------------------GC-CCG-G-C-CUU
BPgV_PDB34_KC796093-1-10457-10557            UUC----CGG-C-AAGG-UGCGC-C-G-G-GGGGGCCU------------UCAC--------GG-GCCCUUCU--A-GCG--CA-G-GGG---UUU-GAG-ACA----CCC-C-CCG------CC-CC-A-CUC-CUU------------------------------CCCAGG-----------------GUUGGCAA--------------------C-CUG-G-G-UCG
BpgV_PDB76_KC796084-1_10117-10214_xr1        GUA----AGG-C-AAGG-UGUGC-A-C-U-GGCAGCCU------------UAAC--------GG-GCUGUUGG---GGCG--CA-G-GGG---CUU-GAG-CAC----CCC-C-CUU------CC-CC-A-CUC-CCAGC----------------------------GGG--------------------GCUUGGCAA-----------------------C-C-C-UGC
BPgV_PDB76_KC796084-1_10385-10487_xr3        GUU-----AG-C-AAGG-CGCUGUCAU-G-GUGCUUC-------------UAAC---------G-AGGCAUGA--GGCAG--CG-G-GGG---CGU-GAG-AAC----CCC-C-CU--U----CC-CC-A-CUC-CGGCG----------------------------UGUAG------------------AUUGGCAAUC--------------------UUG-C-G-CUG
BPgV_PDB76_KC796084-1_10261-10376_xr2        ACU--U--GG-C-AAGG-CACCC---C-CUGGCUAGUGGCUGC---ACUUAACUGA-GUGGCCGGCUAGUCGG--U-GGG--UG-G-GGG---CAC-GUG-ACU----CCC-C-CU--U----CC-CC-A-CAC-CGGC-----------------------------GGCG-------------------GCCGUAAAA----------------------CG-C-C-CCC
BPgV_PDB620_NC_038435-1_10545-10647          UCC----UGG-C-AAGG-GCUAC-U-C-U-GAAAGCUGC-----------UAAC-------GUG-GUGAUCGG--C-GUA--GC-G-GGG---CGU-GAG-GAA----CCC-C-CCA------CC-CC-A-CUC-CUG------------------------------GGUCAG-----------------CUUGGUAA--------------------C-UGG-C-C-CAG
BPgV_PDB1698_NC_038434-1_10238-10344         GGU--A-CGG-C-AACA-CAGGGU--G-C-CC------------GUAACGUCUCG--------------GGGC---AUUC--UG-G-GGU---AUU-GAC-GAC----ACC-C-CCG-U----UG-UG-A-GUC-AGGGGA---------------------------GGGCCAC----------------AAAGCGUCUCGC---------------GU-GGU-U-C-UCC
BPgV_KC796079-1_10155-10253_xr1              GUA----AGG-C-AAGG-UGUG----CAC-UGGCAGCCU-----------UAAC--------GGG-CUGUUGGG--A--CA-CA-G-GGG---CUU-GAG-CAC----CCC-C-CUU------CC-CC-A-CUC-CCAGC----------------------------GGGG-------------------CUUGGCAA-----------------------CC-C-C-CCU
RPgV_NC_021154-1_10821-10910_xr1             UUG----GGG-C-A-GG-CACG----------------------------CUUG------------------------CG--UG-G-GGG---AGU-UGC-GCC----CCC-C-CCC------AG-CC-A-GCA-CUCGUCAUGAUGUGUC-----------------GGAUGCC----------------AGUGAUA--------------------GG-CAG-C-C-UCC
EPgVD_Theiler_NC_038433-1_10245-10317_xr1    UCC--A-AGG-C-AACA-GGC-----------------------------UUCG-------------------------G--CC-G-GGG--GAGU-AGC-GCC----CCC-C-CCU-U----UG-UG-A-GCU-CGUAA----------------------------CCCC-------------------CUUUU--------------------------GG-G-G-CUG
EPgVD_Theiler_NC_038433-1_10385-10449_xr2    GCC----CGG-C-AAGG-U-------------------------------UAAC-----------------------------A-G-GGG--GAGU-AGU-GCC----CCC-C-CCG------CC-CC-A-ACU-CGGGUA---------------------------GCG--------------------CGUA----------------------------C-G-C-UCG
RPgV_GBVD_NC_030291-1_9373-9440              ACA----UGG-C-AAGG-GUGC----------------------------UUCG------------------------GC--AU-G-GGG--GAGU-AGC-GUC----CCU-C-CCA------CC-CC-A-GCG----------------------------------AGGCU------------------GCA---------------------------AGC-C-U-AUC
BPgV_HM047196-1_9710-9781                    ACC----UGG-C-AACA-GCCCC----------------------------GUC-----------------------GGG--GC-G-GGG--GAGU-AGC-GCC----CCC-C-CCA-G----UG-UG-A-GCG-A--------------------------------GGUGG------------------GAAA--------------------------CCA-C-C-UAG
BPgV_PDB1715_NC_038437-1_9683-9754           CAA----GGG-C-AAGG-UGUCU---------------------------UGCG-----------------------AGA--CA-G-GGG---CUU-AAC-GCA----CCC-C-CCC------CC-CC-A-GUG-AG-------------------------------GGGGG------------------CUGA--------------------------UCC-C-C-CAC

Hepacivirus
GBVB_NC_001655-1_9095-9165*                  CAG----CGG-C-AACA-GGG-----------------------------GAGA-------------------------C--CC-C-GGG---CUU-AAC-GAC----CCC-G-CCG-A----UG-UG-A-GUU-UGGC-----------------------------GACCA------------------UGG---------------------------UGGAU-C-AGA
HCGu_NC_031950-1_10278-10351*                CAA--U--GG-C-AACA-GCACU--------------------------CUCUUA----------------------GGU--GC-G-GGG---UAU-GGC-GAA----CCC-C-CC--AA---UG-UG-A-GCU-CCUCCCCGGAUGGGGCGG-------------------------------------------------------------------------------
HCK_NC_038430_9396-9493*                     UUC----GGG-C-AAGG-CAUGCG--A-G-A----------------UAAAAAGGG--------------UCU---CGUA--UG-A-GGG---CGU-GGC-AAC----CCC-U-CCC------CC-CC-A-GCU-GCGG-----------------------------CGGCAC-----------------AAAAGCGUCUCGC---------------G-UGU-C-G-UCG
HCP_NC_040815-1_8489-8574                    UAG----CGG-C-AGGG-AGCAGG-----------------------GUAGACCAA--------------------CCUG--CA-G-GGG---CUU-GAC-GAC----CCC-C-CCG------UC-CCGA-GUC-AGCCAG------------G--------------AGGC------------------AGAAGC-------------------------GAC-U-C-GCG
RHVI_NC_038428-1_8741-8812                   ACA----CUG-C-AACA-GGG-----------------------------GAAA-------------------------C--CC-G-GGG---AUU-UCC-GAU----CCC-C-CAG-A----UG-UG-A-GGA-GGCU-----------------------------GGUUG------------------CCUAA-------------------------CAA-C-C-UGU
HCF_MN242370_8677-8777_xr1                   AAG----GGG-C-AGGA-UAUGGA--C------------------AGAACCGUCUCGG---------------G---UCCAGA-A-G-GGG---CUU-GGC-GAC----CCC-C-CCC-A----UC-CG-A-GCCACCCC-----------------------------UCUAGG------------------AAGACCGUCUCGG--------------C-CUA-G-A-GGG
RtMc_HCV_KY370094-1_8363-8469_!!             UGA----GGG-C-AGGA-UAGGCCAAG-A---------------------AUCGUC----------------UC-GAGGCUGAUU-G-GGG---CUU-CAC-GCA----CCC-C-CCC-A----UC-CG-A-GUG-CCU------------------------------CUCAUCUUCC-------------AAAACCGUCUCGG-----------GGGAG-AUG-A-G-ACC
RHVE_NC_021153-1_8663-8762_!!                CAG----GGG-C-AGAG-GCCGGU--U-G------------------AUUAUCAUUG--------------CA---GCCG--UA-G-GGG---CUU-GGC-GAC----CCC-C-CCC-C----CU-CG-A-GCC-AGCC-----------------------------UUUCAAC----------------AAAACCGUCUCGG--------------GU-UGG-A-A-GGC
RHV_KX905133-1_9479-9553_!!                  AAA----GGG-C-AGCG-GUGCC---------------------------GGAC-----------------------GGC--AUUG-GGG---CUU-GGC-GAC----CCC-C-CCC-A----CG-CG-A-GCU----------------------------------ACCCACC----------------AUU------------------------GG-UGG-G-U-UCC
SifHV_MH824541_8631-8725_xr1_!!              AAG----GGG-C-AGCC-AUGCCG--C------------------AGAACCGUCUCGG---------------G---CGGC--AA-G-GGG---CUU-AGC-GAC----CCC-C-CCC-U----GG-CG-A-GCUGUAUGAGUGUGAUAAGGGCGACAUAGCG----------------------------------------------------------------------

#=GC SS_cons                                 -------------(((((((((((--(-(-(((((((((((((--------------)))))))-))))))))---))))--))-(((((------------------)))))---------)))-))----------------------------------------((((((((((((---------------------------------)))))))-)))-)-)----
#=GC SS_cons_1(Pk1)                          -------(((--------------------------------------------------------------------------------------------------------)))---------------------------------------------------------------------------------------------------------------------
#=GC SS_cons_2(Pk2)                          -------------------------------------------------------------------------------------------------(((-------------------------------)))--------------------------------------------------------------------------------------------------
```
