# Supplementary material for: A New Subclass of Exoribonuclease-Resistant RNA Found in Multiple Genera of Flaviviridae
Source: mBio. 2020 Sep 29;11(5):e02352-20. doi: 10.1128/mBio.02352-20 (PMC7527734; doi:10.1128/mBio.02352-20)
Supplement: TABLE S2 [file mBio.02352-20-st002.pdf]

Table S2: General information about subclass 1b sequence alignment

Alignment Name	GenBank or RefSeq ID	xrRNA Context	Virus Name	Total ssRNA length (nt)	xrRNA Length (nt)	Overall ssRNA Location	Genera Classification
TABV_AF346759-1_10222-10300_xr*+	AF346759.1	xr1	Tamana Bat Virus, complete genome	10428	79	10222-10300	Divergent Flavivirus
TABV_AF346759-1_10305-10380_xr2	AF346759.1	xr2	Tamana Bat Virus, complete genome	10428	76	10305-10380	Divergent Flavivirus
CXFV_NC_008604-2_10246-10326_xr1+	NC_008604.2	xr1	Culex flavivirus, complete genome	10837	81	10246-10326	Flavivirus
CXFV_NC_008604-2_10365-10445_xr2+	NC_008604.2	xr2	Culex flavivirus, complete genome	10837	81	10365-10445	Flavivirus
CXFV_NC_008604-2_10478-10552_xr3+	NC_008604.2	xr3	Culex flavivirus, complete genome	10837	81	10478-10552	Flavivirus
CXFV_NC_008604-2_10598-10675_xr4+	NC_008604.2	xr4	Culex flavivirus, complete genome	10837	81	10598-10675	Flavivirus
CTFV_HE574574-1_10178-10264_xr2	HE574574.1	xr2	Culex theileri flavivirus RP-2011 gene for viral polyprotein, genomic RNA, isolate 178	10538	87	10178-10264	Flavivirus
CTFV_HE574574-1_10280-10359_xr3	HE574574.1	xr3	Culex theileri flavivirus RP-2011 gene for viral polyprotein, genomic RNA, isolate 178	10538	80	10280-10539	Flavivirus
CTFV_HE574574-1_10406-10490_xr4+	HE574574.1	xr4	Culex theileri flavivirus RP-2011 gene for viral polyprotein, genomic RNA, isolate 178	10538	85	10406-10490	Flavivirus
QBV_NC_012671-1_10245-10332_xr1+	NC_012671.1	xr1	Quang Binh Virus, complete genome	10865	88	10245-10332	Flavivirus
QBV_NC_012671-1_10344-10427_xr2+	NC_012671.1	xr2	Quang Binh Virus, complete genome	10865	84	10344-10427	Flavivirus
QBV_NC_012671-1_10490-10575_xr3+	NC_012671.1	xr3	Quang Binh Virus, complete genome	10865	86	10490-10575	Flavivirus
QBV_NC_012671-1_10620-10703_xr4+	NC_012671.1	xr4	Quang Binh Virus, complete genome	10865	84	10620-10703	Flavivirus
YNCxFV_NC_021069-1_10244-10331_xr1+	NC_021069.1	xr1	Mosquito flavivirus isolate LSFlaiV-A20-09, complete genome	10865	88	10244-10331	Flavivirus
YNCxFV_NC_021069-1_10343-10426_xr2+	NC_021069.1	xr2	Mosquito flavivirus isolate LSFlaiV-A20-09, complete genome	10865	84	10343-10426	Flavivirus
YNCxFV_NC_021069-1_10489-10576_xr3+	NC_021069.1	xr3	Mosquito flavivirus isolate LSFlaiV-A20-09, complete genome	10865	88	10489-10576	Flavivirus
YNCxFV_NC_021069-1_10621-10704_xr4+	NC_021069.1	xr4	Mosquito flavivirus isolate LSFlaiV-A20-09, complete genome	10865	84	10621-10704	Flavivirus
CTFVtur3_KX652378-1_10323-10409_xr1+	KX652378.1	xr1	Mosquito flavivirus isolate 599, complete genome	10697	87	10323-10409	Flavivirus
CTFVtur3_KX652378-1_10424-10505_xr2	KX652378.1	xr2	Mosquito flavivirus isolate 599, complete genome	10697	82	10424-10505	Flavivirus
CTFVtur3_KX652378-1_10552-10636_xr3	KX652378.1	xr3	Mosquito flavivirus isolate 599, complete genome	10697	85	10552-10636	Flavivirus
CFAV_NC_001564-2_10173-10247_xr1+	NC_001564.2	xr1	Cell fusing agent virus strain Galveston, complete genome	10682	75	10173-10247	Flavivirus
CFAV_NC_001564-2_10260-10335_xr2+	NC_001564.2	xr2	Cell fusing agent virus strain Galveston, complete genome	10682	76	10260-10335	Flavivirus
CFAV_NC_001564-2_10555-10632_xr3_!!	NC_001564.2	xr3	Cell fusing agent virus strain Galveston, complete genome	10682	78	10555-10632	Flavivirus
AEFV_NC_012932-1_10496-10574_xr1_+	NC_012932.1	xr1	Aedes flavivirus genomic RNA, complete genome, strain: Narita-21	11064	79	10496-10574	Flavivirus
AEFV_NC_012932-1_10634-10712_xr2_+	NC_012932.1	xr2	Aedes flavivirus genomic RNA, complete genome, strain: Narita-21	11064	79	10634-10712	Flavivirus
AEFV_NC_012932-1_10933-11012_xr3	NC_012932.1	xr3	Aedes flavivirus genomic RNA, complete genome, strain: Narita-21	11064	80	10933-11012	Flavivirus
AEFV_KJ741266-1_10497-10572_xr1	KJ741266.1	xr1	Aedes flavivirus strain Bangkok, complete genome	11063	79	10497-10572	Flavivirus
AnFV_NC_031327-1_10141-10226_xr1	NC_031327.1	xr1	Anopheles flavivirus variant1, complete genome	10588	87	10141-10227	Flavivirus
NIEV_NC_024299-2_10211-10304_xr1	NC_024299.2	xr1	Nienokoue virus isolate B51/CI/2004, complete genome	10878	94	10211-10304	Flavivirus
NIEV_NC_024299-2_10349-10434_xr2	NC_024299.2	xr2	Nienokoue virus isolate B51/CI/2004, complete genome	10878	86	10349-10434	Flavivirus
NIEV_NC_024299-2_10561-10639_xr3	NC_024299.2	xr3	Nienokoue virus isolate B51/CI/2004, complete genome	10878	79	10561-10639	Flavivirus
NIEV_NC_024299-2_10637-10716_xr4	NC_024299.2	xr4	Nienokoue virus isolate B51/CI/2004, complete genome	10878	80	10637-10716	Flavivirus
OCFV_NC_034242-1_10279-10360_xr1	NC_034242.1	xr1	Ochlerotatus caspius flavivirus isolate 1608 polyprotein gene, complete cds	10370	82	10279-10360	Flavivirus
PaRV_NC_027817-1_10318-10401_xr1	NC_027817.1	xr1	Parramatta River virus isolate 92-B115745, complete genome	10893	84	10318-10401	Flavivirus
PaRV_NC_027817-1_10442-10524_xr2	NC_027817.1	xr2	Parramatta River virus isolate 92-B115745, complete genome	10893	83	10442-10524	Flavivirus
PaRV_NC_027817-1_10540-10629_xr3	NC_027817.1	xr3	Parramatta River virus isolate 92-B115745, complete genome	10893	90	10540-10629	Flavivirus
PaRV_NC_027817-1_10757-10848_xr4_!!	NC_027817.1	xr4	Parramatta River virus isolate 92-B115745, complete genome	10893	92	10757-10848	Flavivirus
KRV_NC_005064-1_10358-10439_xr1+	NC_005064.1	xr1	Kamiti River virus, complete genome	11375	82	10358-10439	Flavivirus
KRV_NC_005064-1_10954-11041_xr2+	NC_005064.1	xr2	Kamiti River virus, complete genome	11375	88	10954-11041	Flavivirus
MECDV_NC_027819-1_10811-10887_xr1_!!	NC_027819.1	xr1	Mercadeo virus isolate ER-M10, complete genome	10938	77	10811-10887	Flavivirus
XFV_NC_034017-1_10365-10443	NC_034017.1	xr1	Xishuangbanna aedes flavivirus, complete genome	10884	79	10365-10443	Flavivirus
MFV_NC_034204-1_10386-10482_!!	NC_034204.1	xr1	Menghai flavivirus isolate MHAedFV1, complete genome	10386	97	10386-10482	Flavivirus
ALFv_KJ412989_202-279_xr1	KJ412989	xr1	Flavivirus Abbeylake 3' UTR	428	78	202-279	Flavivirus
EPgV_NC_020902-1_10836-10900_xr1	NC_020902.1	xr1	Equine Pegivirus 1 isolate C0035, complete genome	11197	65	10836-10900	Pegivirus
EPgV_NC_020902-1_10957-11019_xr2	NC_020902.1	xr2	Equine Pegivirus 1 isolate C0035, complete genome	11197	63	10957-11019	Pegivirus
EPgV_MF438044-1_10714-10779_xr1	MF438044.1	xr1	Equine pegivirus strain LW/2016 polyprotein gene, complete cds	11075	66	10714-10779	Pegivirus
EPgV_MF438044-1_10834-10896_xr2	MF438044.1	xr2	Equine pegivirus strain LW/2016 polyprotein gene, complete cds	11075	63	10834-10896	
HPgV_AF070476-1_9015-9094	AF070476.1	xr1	GB virus C variant troglodytes, complete genome	9250	80	9015-9094	Pegivirus
SPgV_NC_001837-1_9441-9518_GBVA*	NC_001837.1	xr1	Hepatitis GB virus A, complete genome	9550	78	9441-9518	Pegivirus
SPgVkob_KF234530-1_9178-9267	KF234530.1	xr1	Simian pegivirus isolate SPgVkob_OB23 polyprotein precursor, gene, complete cds	9322	90	9178-9267	Pegivirus
HPgV_KT166442-1_9100-9186	KT166442.1	xr1	GB virus C isolate GBV-C_agmMal_seqID_10 putative polyprotein gene, complete cds	9239	87	9100-9186	Pegivirus
SPgVkrc_NC_024377_9230-9314	NC_024377.1	xr1	Simian pegivirus isolate SPgVkrc_RC08 polyprotein precursor, gene, complete cds	9525	85	9230-9314	Pegivirus
SPgVkrtg_KF234529-1_9142-9233	KF234529.1	xr1	Simian pegivirus isolate SPgVkrtg_RT11 polyprotein precursor, gene, complete cds	9276	92	9142-9233	Pegivirus
HPgV_AB008335-1_9096-9189	AB008335.1	xr1	Hepatitis GB virus C genomic RNA, complete sequence, strain: K3732	9391	94	9096-9189	Pegivirus
HPgV_NC_001710-1_9098-9182	NC_001710.1	xr1	GB virus C/Hepatitis G virus, complete genome	9392	94	9095-9188	Pegivirus
HPgV_AB018667-1_8931-9024	AB018667.1	xr1	Hepatitis G virus genomic RNA for polyprotein, complete cds	9228	94	8931-9024	Pegivirus
HPgV2_NC_027998-2_9532-9636*	NC_027998.2	xr1	Human pegivirus 2 isolate UC0125.US polyprotein gene, complete cds	9867	104	9532-9636	Pegivirus
BPgV_PDB34_KC796093-1-10457-10557	KC796093.1	xr1	Bat pegivirus isolate PDB-34.1 polyprotein gene, complete cds	10580	101	10457-10557	Pegivirus
BpgV_PDB76_KC796084-1_10117-10214_xr1	KC796084.1	xr1	Bat pegivirus isolate PDB-76.1 polyprotein gene, complete cds	10487	98	10117-10214	Pegivirus
BPgV_PDB76_KC796084-1_10385-10487_xr3	KC796084.1	xr3	Bat pegivirus isolate PDB-76.1 polyprotein gene, complete cds	10487	103	10385-10487	Pegivirus
BPgV_PDB76_KC796084-1_10261-10376_xr2	KC796084.1	xr2	Bat pegivirus isolate PDB-76.1 polyprotein gene, complete cds	10487	117	10261-10376	Pegivirus
BPgV_PDB620_NC_038435-1_10545-10647	NC_038435.1	xr1	Pegivirus G isolate PDB-620 polyprotein gene, complete cds	10767	103	10545-10647	Pegivirus
BPgV_PDB1698_NC_038434-1_10238-10344	NC_038434.1	xr1	Pegivirus F isolate PDB-1698 polyprotein gene, complete cds	10465	107	10238-10344	Pegivirus
BPgV_KC796079-1_10155-10253_xr1	KC796079.1	xr1	Bat pegivirus isolate PDB-99 polyprotein gene, complete cds	10392	99	10155-10253	Pegivirus
RPgV_NC_021154-1_10821-10910_xr1	NC_021154.1	xr1	Rodent pegivirus isolate CC61, complete genome	11279	90	10821-10910	Pegivirus
EPgVD_Theiler_NC_038433-1_10385-10449_xr1	NC_038433.1	xr1	Theiler's disease-associated virus isolate HorseA1_serum, complete genome	10479	65	10385-10449	Pegivirus
EPgVD_Theiler_NC_038433-1_10385-10449_xr2	NC_038433.1	xr2	Theiler's disease-associated virus isolate HorseA1_serum, complete genome	10479	73	10245-10317	Pegivirus
RPgV_GBVD_NC_030291-1_9373-9440	NC_030291.1	xr1	GB virus D strain 68 polyprotein precursor, gene, complete cds	9630	68	9373-9440	Pegivirus
BPgV_HM047196-1_9710-9781	HM047196.1	xr1	Bat GB-like virus JFD-2011 strain BtGBV/S60/Eid_hel/GH/2009 polyprotein gene, complete cds	9849	72	9710-9781	Pegivirus
BPgV_PDB1715_NC_038437-1_9683-9754	NC_038437.1	xr1	Pegivirus I isolate PDB-1715 polyprotein gene, complete cds	9777	72	9683-9754	Pegivirus
RPgV_NC_021154-1_11072-11138_xr2	NC_021154.1	xr2	Rodent pegivirus isolate CC61, complete genome	11279	67	11072-11138	Pegivirus
NRPeV_NC_025677-1_12526-12618_CP	NC_025677.1	xr1	Norway rat pestivirus isolate NrPV/NYC-D23 polyprotein gene, complete cds	12983	93	12526-12618	Pestivirus
RPeV_KY370101-1_12622-12718_xr2_CP	KY370101.1	xr2	Rodent pestivirus isolate RtNn-PestV/HuB2014 polyprotein gene, complete cds	13,220	102	12622-12718	Pestivirus
APPeV_NC_038964-1_11102-11187_CP	NC_038964.1	xr1	Porcine pestivirus 1 strain 000515 polyprotein mRNA, complete cds	11276	86	11102-11187	Pestivirus
RPeV_KY370100-1_12385-12488_xr1	KY370100.1	xr1	Rodent pestivirus isolate RtAp-PestV/JL2014 polyprotein gene, complete cds	12768	104	12385-12488	Pestivirus
BtPeV_MH282908-1_11498-11573_xr1	MH282908.1	xr1	Bat pestivirus BtSk-PestV-1/GX2017 polyprotein gene, complete cds	11921	76	11498-11573	Pestivirus
BtPeV_MH282908-1_11626-11697_xr2	MH282908.1	xr2	Bat pestivirus BtSk-PestV-1/GX2017 polyprotein gene, complete cds	11921	72	11626-11697	Pestivirus
BtPeV_MH282908-1_11716-11812_xr3	MH282908.1	xr3	Bat pestivirus BtSk-PestV-1/GX2017 polyprotein gene, complete cds	11921	97	11716-11812	Pestivirus
GBVB_NC_001655-1_9095-9165_CP	NC_001655.1	xr1	Hepatitis GB virus B, complete genome	9399	71	9095-9165	Hepacivirus
HCGu_NC_031950-1_10278-10351_CP	NC_031950.1	xr1	Guereza hepacivirus, complete sequence	10471	74	10278-10351	Hepacivirus
HCK_NC_038430-1_9396-9493	NC_038430.1	xr1	Hepacivirus K, complete genome	9609	98	9396-9493	Hepacivirus
HCP_NC_040815-1_8489-8574	NC_040815.1	xr1	Hepacivirus P isolate RHV-GS2015, complete genome	8684	86	8489-8574	Hepacivirus
RtMc_HCV_KY370094-1_8363-8469_!!	KY370094.1	xr1	Rodent hepacvirus isolate RtMc-HCV/Tibet2014 polyprotein gene, complete cds	8575	107	8363-8469	Hepacivirus
RHV_KX905133-1_9479-9553_!!	KX905133.1	xr1	Rodent hepacivirus isolate rn-1 polyprotein gene, complete cds	9656	75	9479-9553	Hepacivirus
RHVE_NC_021153-1_8663-8762_!!	NC_021153.1	xr1	Rodent hepacivirus, complete genome	8879	100	8663-8762	Hepacivirus
RHVI_NC_038428-1_8741-8812	NC_038428.1	xr1	Hepacivirus I, complete genome	8883	72	8741-8812	Hepacivirus
HCF_MN242370_8677-8777_xr1	MN242370	xr1	Hepacivirus F isolate MgHV3, complete genome	8836	101	8677-8777	Hepacivirus
SifHV_MH824541_8631-8725 _xr1_!!!	MH824541	xr1	Sifaka hepacivirus strain SifHV_H2-L41, complete genome	8729	95	8631-8725	Hepacivirus

Pegivirus
