# Supplementary material for: A New Subclass of Exoribonuclease-Resistant RNA Found in Multiple Genera of Flaviviridae
Source: mBio. 2020 Sep 29;11(5):e02352-20. doi: 10.1128/mBio.02352-20 (PMC7527734; doi:10.1128/mBio.02352-20)
Supplement: TABLE S3 [file mBio.02352-20-st003.pdf]

Table S3: *In vitro* transcribed subclass 1b construct info

Name	Sequence
**APPeV_NC_038964-1_11102-11187_CP**	5’-TAATACGACTCACTATAGGAGGCGTCGAGTAGACGCCAAATGAAAGGGGCAAGTGGCCGTATAGGCTGGGGCGATCGCCGTACCCCCCCTTTACCAGGCGCCTCAACCCCATGTACCATGGGGTTGAAAGGAGTCGAGTAGACTCCAACAAAAGAAACAACAACAACAAC-3
**RPeV_KY370101-1_12622-12718_xr2_CP**	5’-TAATACGACTCACTATAGGAGGCGTCGAGTAGACGCCAAAAAAGGCAGGGAGAGGCTTAAGAACCGTCTCGGGAGCCTCTTGGGGCTTGACGAACCCCCCAACCCGAGTCAAGTCCTTCAACAGTACCGTTTCGAGGACAAAAAGGAGTCGAGTAGACTCCAACAAAAGAAACAACAACAACAAC -3
**NRPeV_NC_025677-1_12526-12618_CP**	5’-TAATACGACTCACTATAGGAGGCGTCGAGTAGACGCCAAAGAATAGGCAGGGAGGAGTCCAAGAACCGTCTCGGGGACTCTTTGGGGCTTGACGAACCCCCCTACCCGAGTCTATATTCAGTGGCTGGAACCGAAAGGAGTCGAGTAGACTCCAACAAAAGAAACAACAACAACAAC-3
**SPgV_NC_001837-1_9441-9518_GBVA_CP**	5’-TAATACGACTCACTATAGGAGGCGTCGAGTAGACGCCAAACTCGAGGCAGCAGCAGACGCAAGTCTGGGGGAAACGATCGCTCCTCCCTCTGCAGATCTCTAGCTCGGATAGAGCGGGAAAGGAGTCGAGTAGACTCCAACAAAAGAAACAACAACAACAAC -3’
**HPgV2_NC_027998-2_9532-9636_CP**	5’-TAATACGACTCACTATAGGAGGCGTCGAGTAGACGCCAAAACAAGGCAGGAGGTGAAGTCAGCTGTACCCACGGCTGGCTGAAACCGGGGCTTGACGACCCCCCCTATCCGAGTTGGGCAAGGTAACATCACGGGTGTGACGACCCCGAAAGGAGTCGAGTAGACTCCAACAAAAGAAACAACAACAACAAC-3
**HCK_NC_038430_9396-9493_CP**	5’-TAATACGACTCACTATAGGCGGCGTCGAGTAGACGCCAAAGGGCAAGGCATGCGAGATAAAAAGGGTCTCGTATGAGGGCGTGGCAACCCCTCCCCCCCAGCTGCGGCGGCACAAAAGCAAAGGAGTCGAGTAGACTCCAACAAAAGAAACAACAACAACAAC-3
**HCGu_NC_031950-1_10278-10351_CP**	5’-TAATACGACTCACTATAGGCGGCGTCGAGTAGACGCCAAACAATGGCAACAGCACTCTCTTAGGTGCGGGGTATGGCGAACCCCCCAATGTGAGCTCCTCCCCGGATGGGGCGGAAAGGAGTCGAGTAGACTCCAACAAAAGAAACAACAACAACAAC-3
**GBVB_NC_001655-1_9095-9165_CP**	5’-TAATACGACTCACTATAGGCGGCGTCGAGTAGACGCCAAACAGCGGCAACAGGGGAGACCCCGGGCTTAACGACCCCGCCGATGTGAGTTTGGCGACCATGGTGGATCAGAAAAGGAGTCGAGTAGACTCCAACAAAAGAAACAACAACAACAAC-3
**PCR Forward Primer**	5’- TAATACGACTCACTATAGGAGGCGTCGAGTAGACG -3’
**PCR Reverse Primer**	5’- GTTGTTGTTGTTGTTTCTTTTGTTGGAGTCTACTCGACTCC -3’
**HCK_GBVB_HCGu Forward Primer**	5’-TAATACGACTCACTATAGGCGGCGTCGAGTAGACG-3’
**FAM Labeled Reverse Transcription Primer**	5’-/5-6FAM/AAAAAAAAAAAAAAAAAAAAAGTTGTTGTTGTTGTTTCTTT-3’

T7 start site

Chemical probing hairpin

XrRNA sequence

FAM labeled primer binding site
