# Supplementary material for: A New Subclass of Exoribonuclease-Resistant RNA Found in Multiple Genera of Flaviviridae
Source: mBio. 2020 Sep 29;11(5):e02352-20. doi: 10.1128/mBio.02352-20 (PMC7527734; doi:10.1128/mBio.02352-20)
Supplement: FIG S2 [file mBio.02352-20-sf002.pdf]

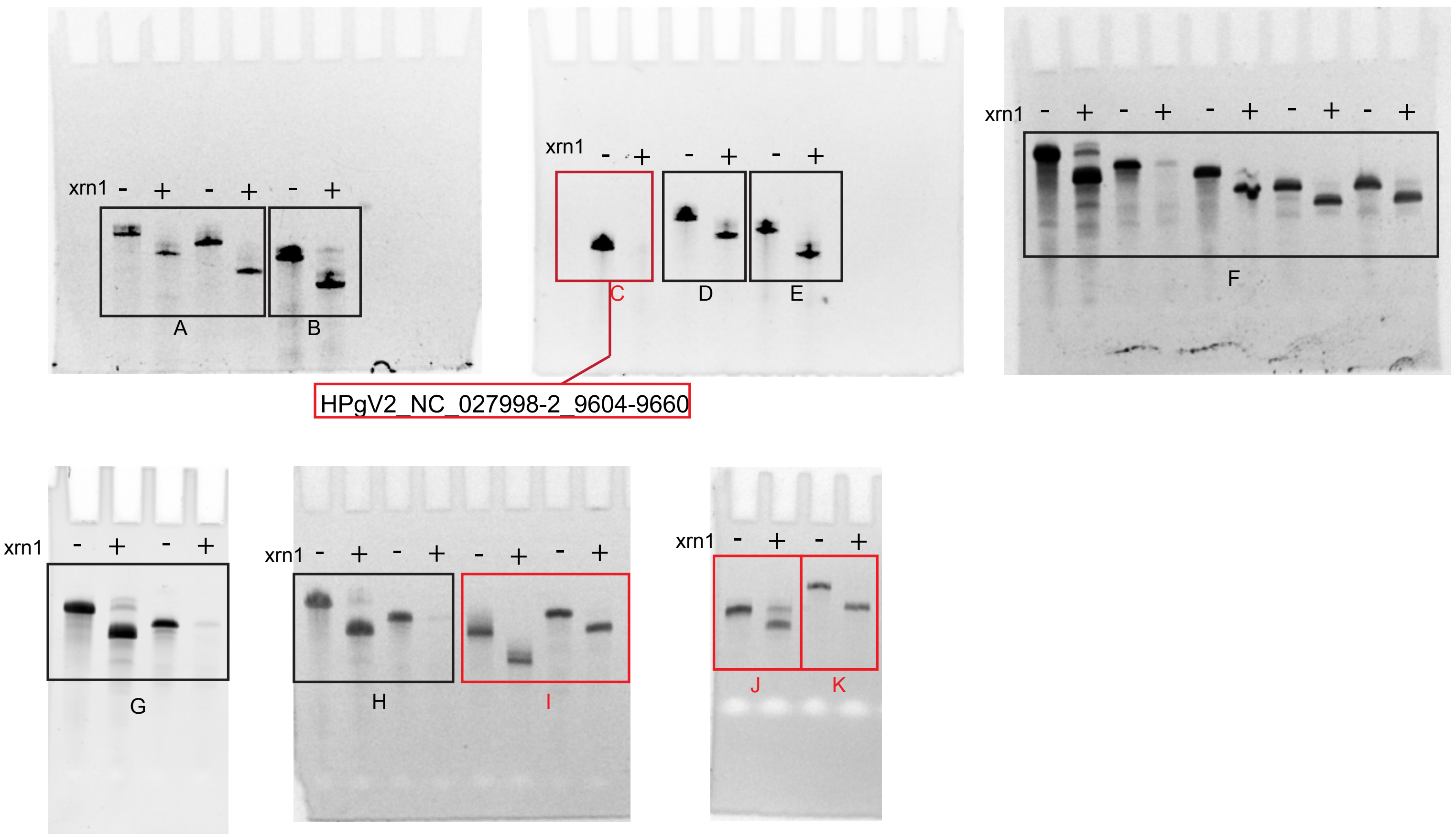

A. Figure 3A: RPeV & APPeV
B. Figure 3B: SPgV
C. HPgV2 (different register not shown in main text)
D. Figure 3B: HPgV2
E. Figure 3A: NRPeV
F. Figure 3C: *Hepacivirus* subclass 1b xrRNA
G. Figure 3B: TABV & negative control
H. Figure 3A: TABV & negative control
I. APPeV & RPeV (replicates not shown in main text)
J. HCP (not shown in main text)
K. GBV-C (not shown in main text)
