# Supplementary material for: A New Subclass of Exoribonuclease-Resistant RNA Found in Multiple Genera of Flaviviridae
Source: mBio. 2020 Sep 29;11(5):e02352-20. doi: 10.1128/mBio.02352-20 (PMC7527734; doi:10.1128/mBio.02352-20)
Supplement: FIG S3 [file mBio.02352-20-sf003.pdf]

A

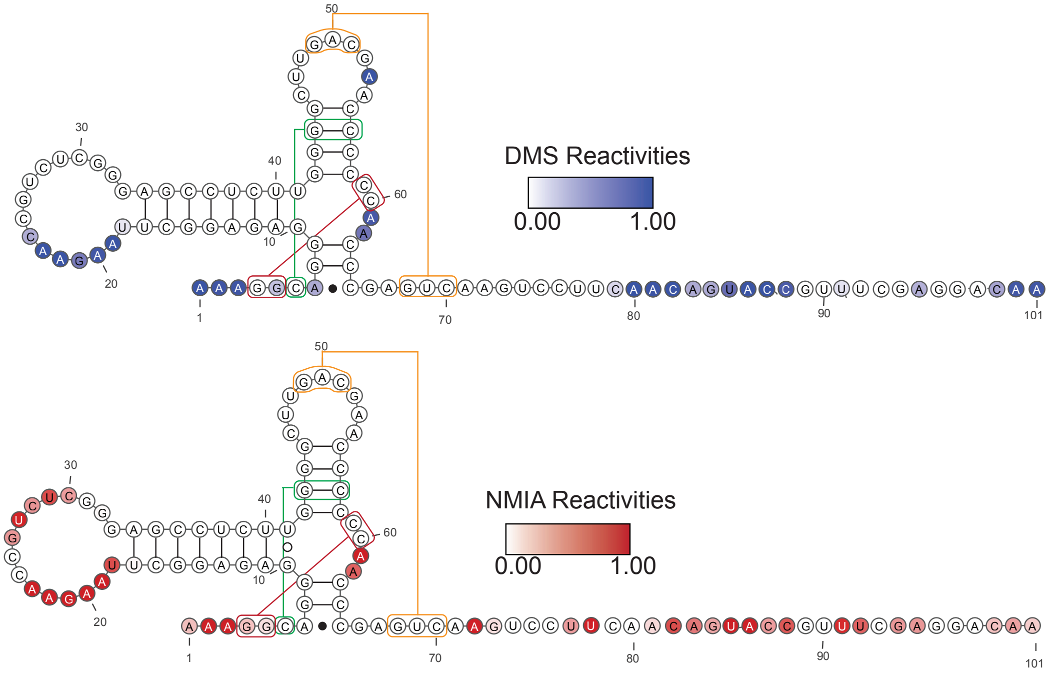

Rodent Pestivirus
(KY370101.1)

B

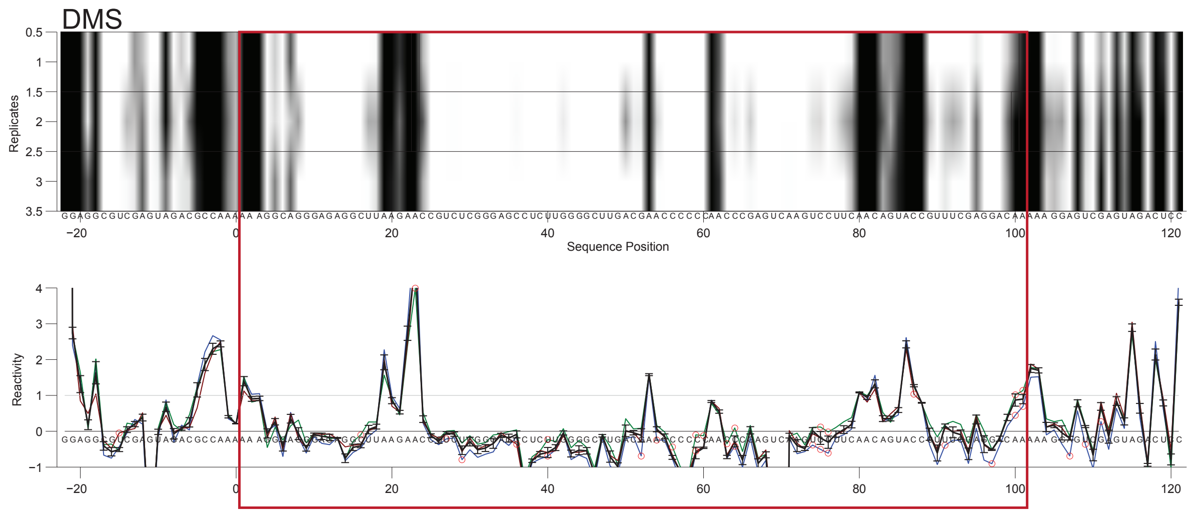

C

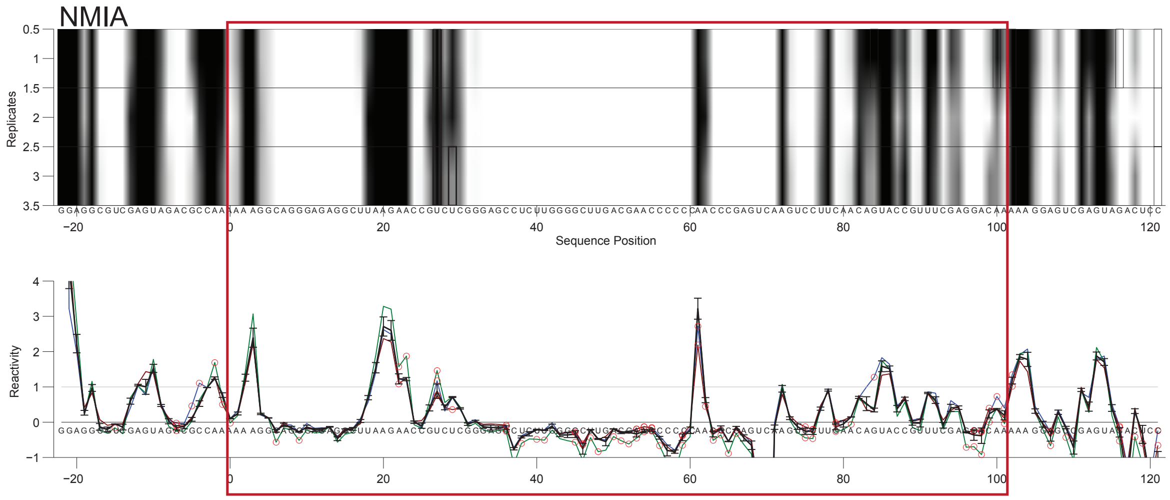

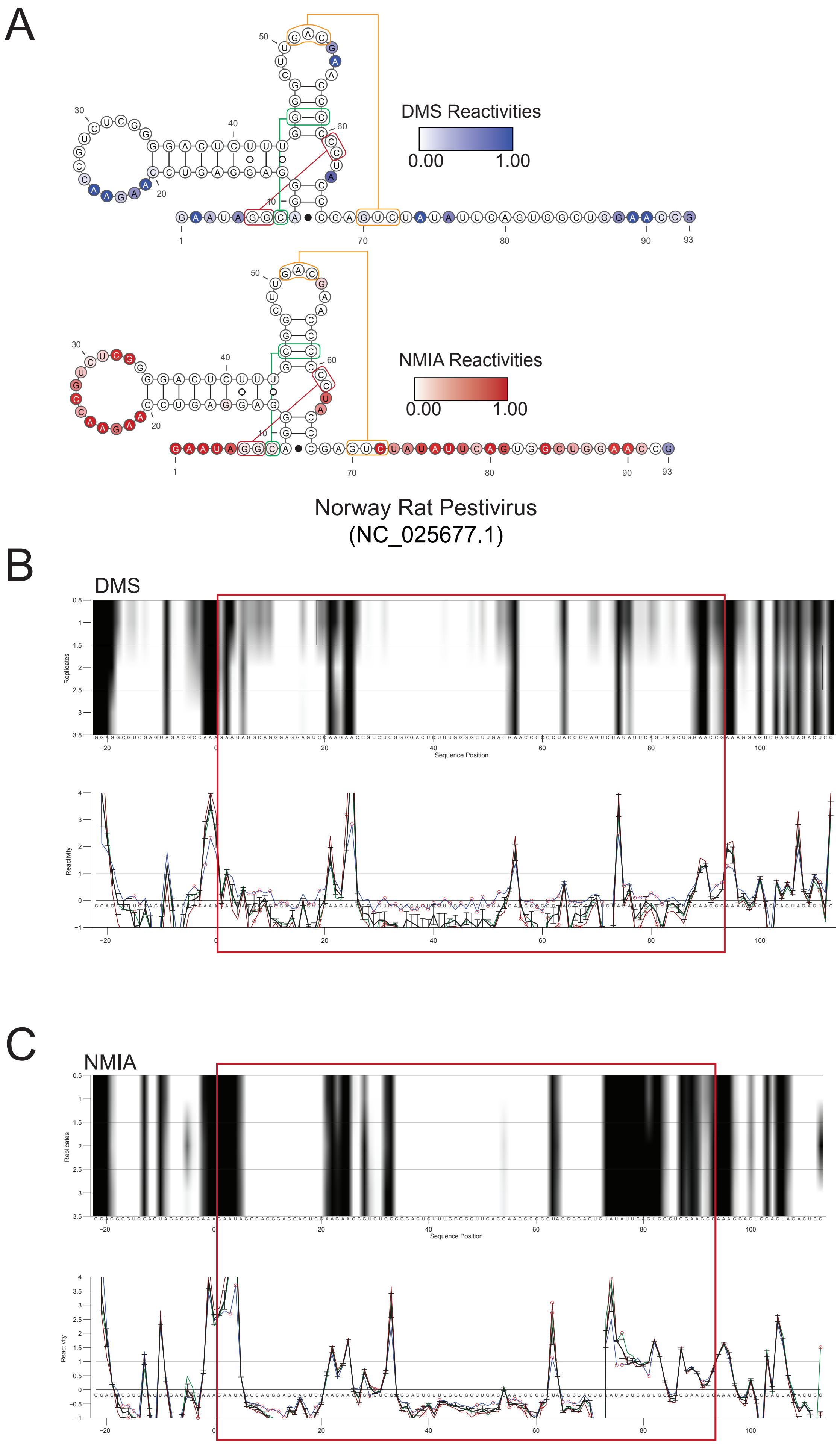
A
DMS Reactivities
0.00
1.00
NMIA Reactivities
0.00
1.00
Norway Rat Pestivirus
(NC_025677.1)
B
DMS
Replicates
Sequence Position
Reactivity
C
NMIA
Replicates
Sequence Position
Reactivity

A

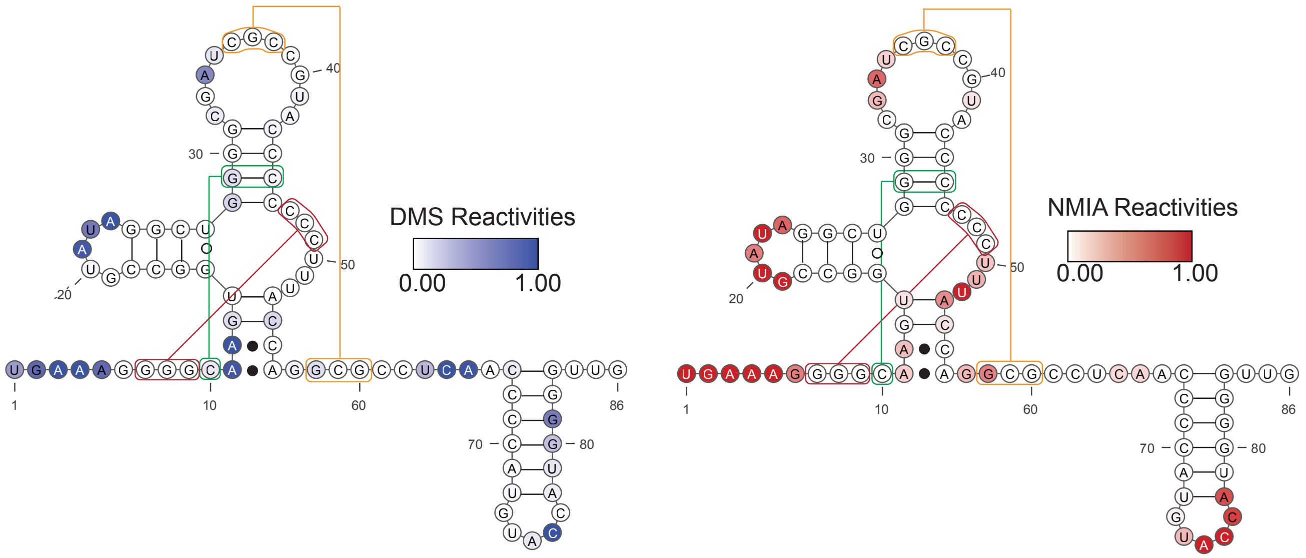

Atypical Porcine Pestivirus
(NC_038964.1)

B

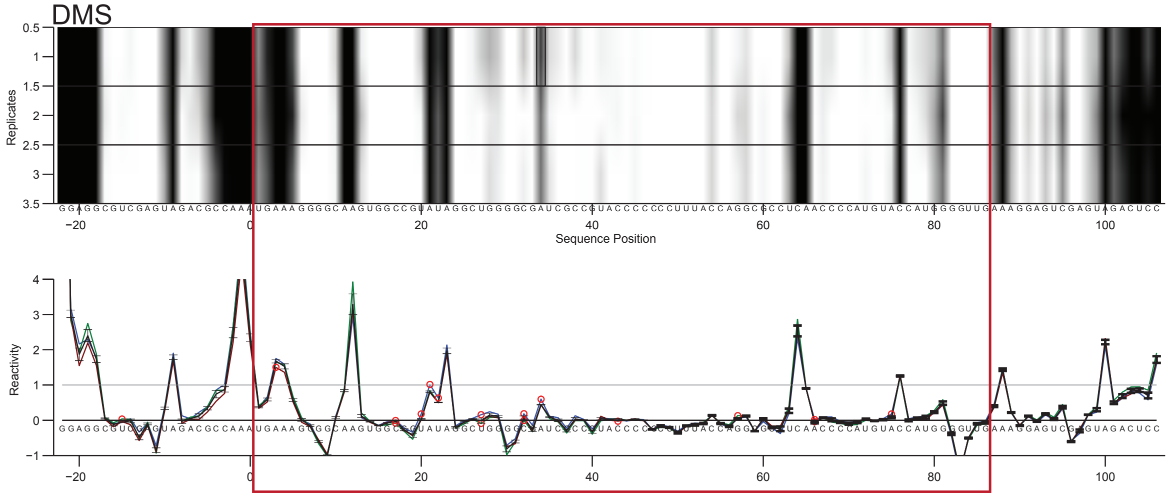

C

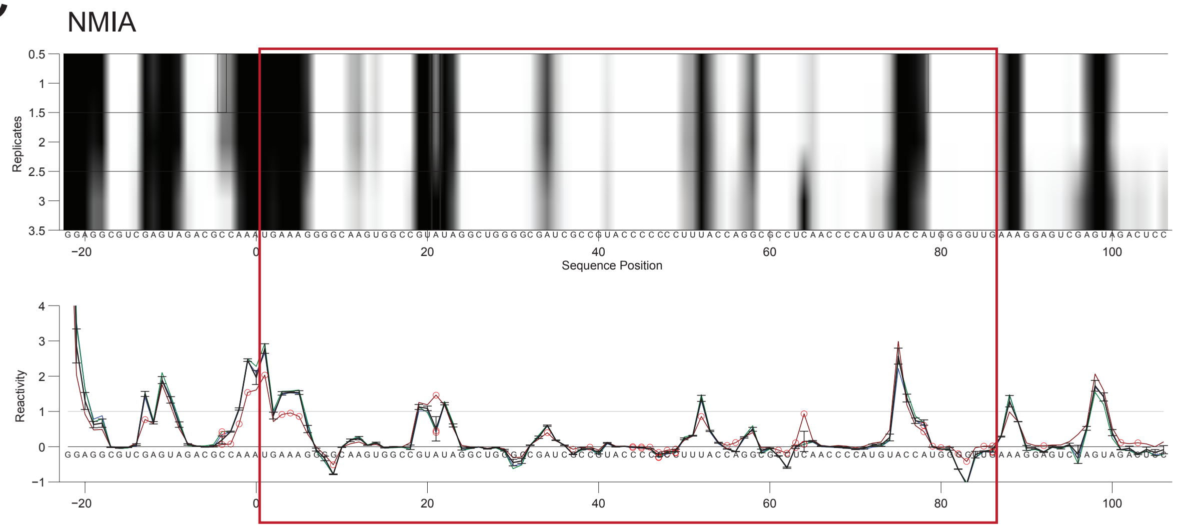

Pegivirus: Simian Pegivirus

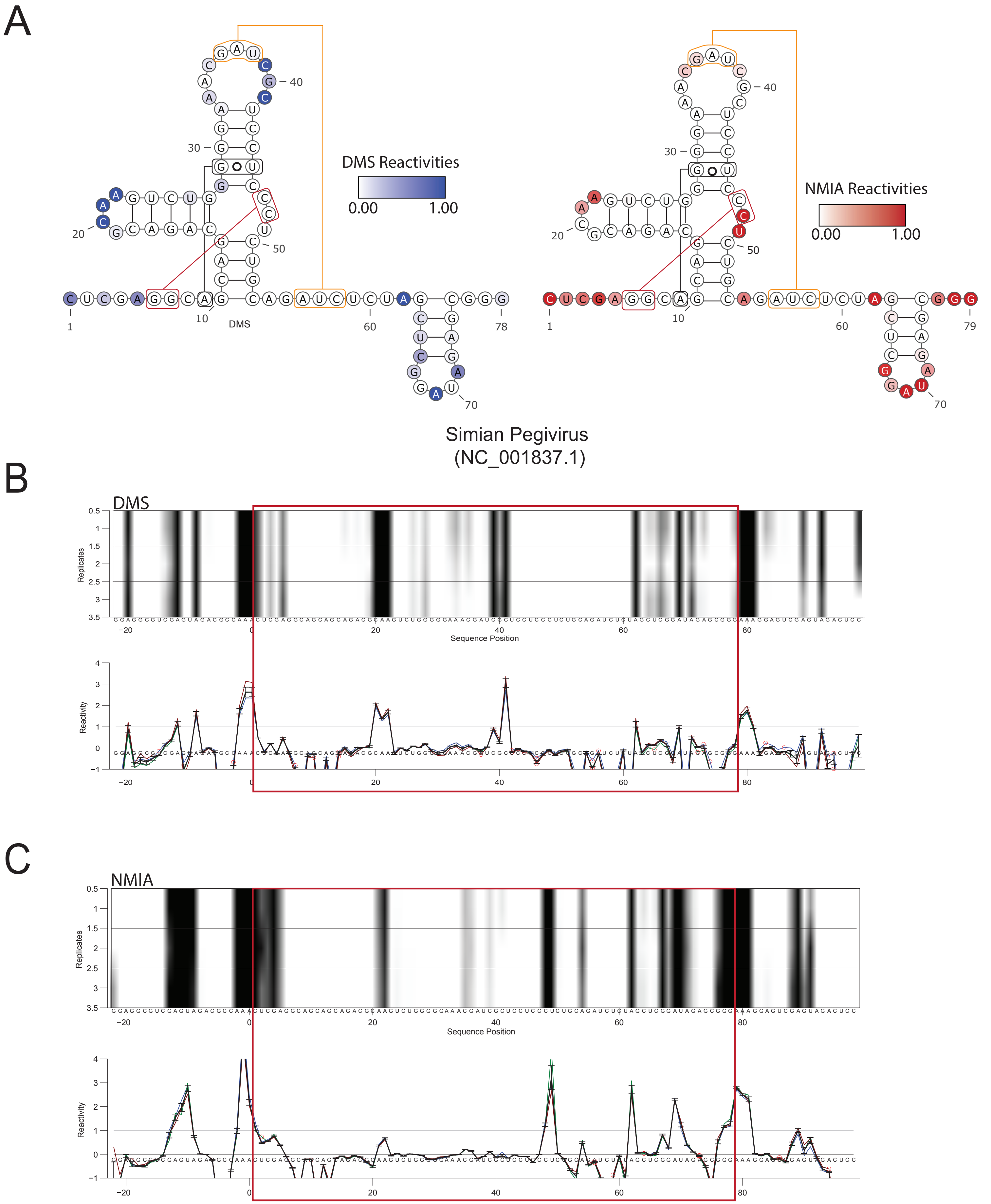

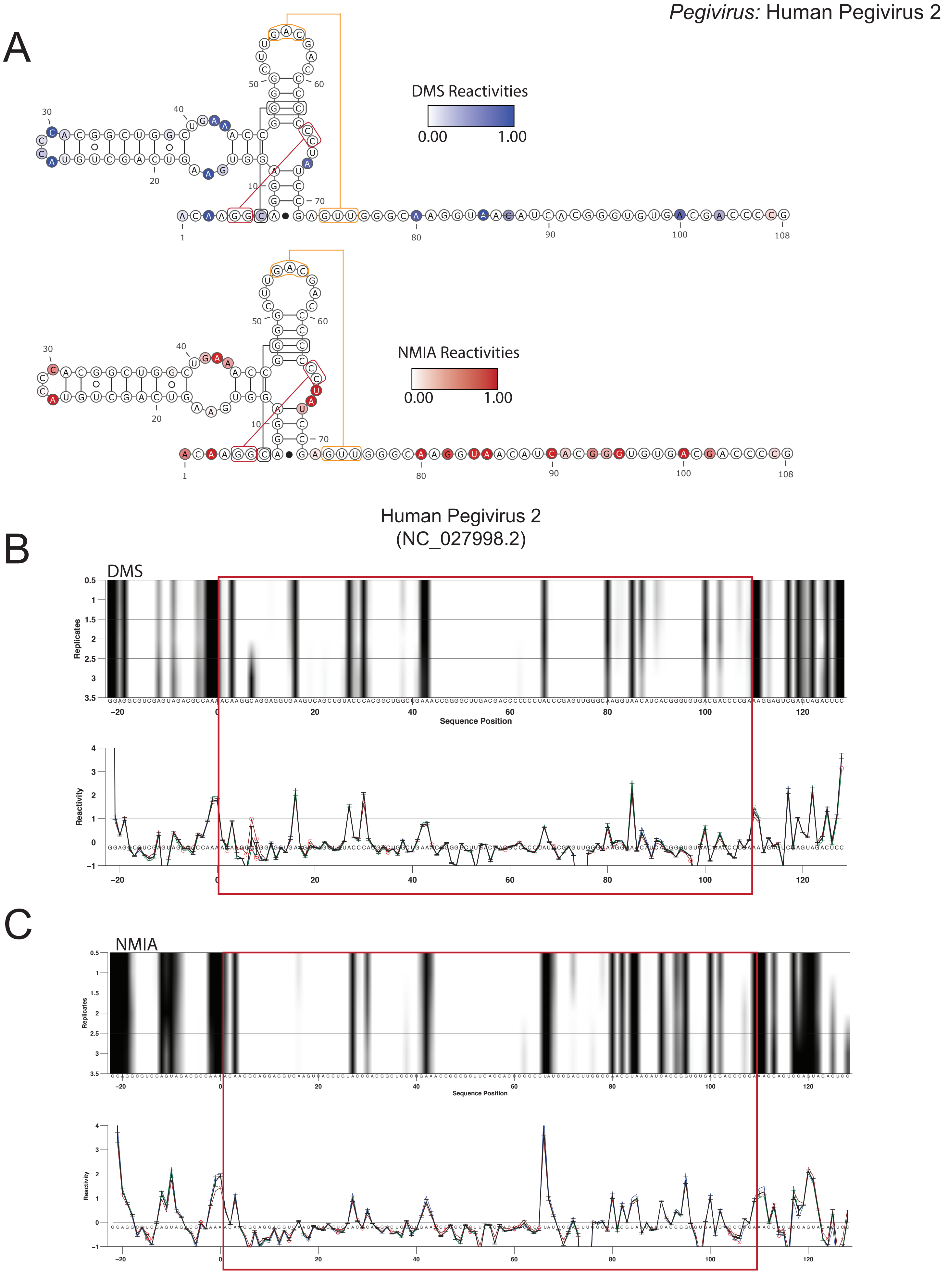

Pegivirus: Human Pegivirus 2
A
DMS Reactivities
0.00
1.00
NMIA Reactivities
0.00
1.00
B
Human Pegivirus 2
(NC_027998.2)
DMS
Replicates
Sequence Position
Reactivity
C
NMIA
Replicates
Sequence Position
Reactivity

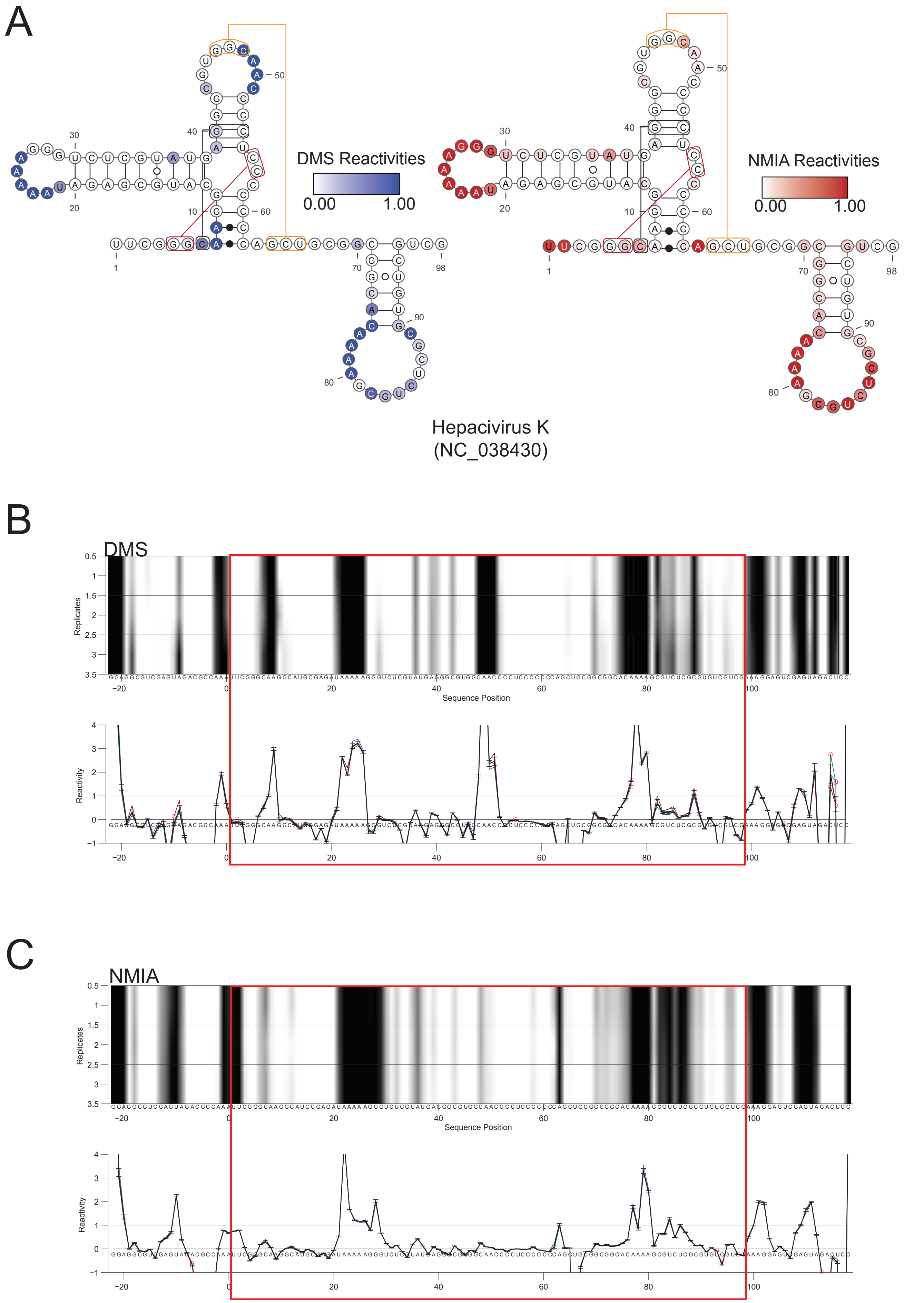
A
DMS Reactivities
0.00
1.00
NMIA Reactivities
0.00
1.00
Hepacivirus K
(NC_038430)
B
DMS
Replicates
Sequence Position
Reactivity
C
NMIA
Replicates
Sequence Position
Reactivity

Hepacivirus: Guereza Hepacivirus

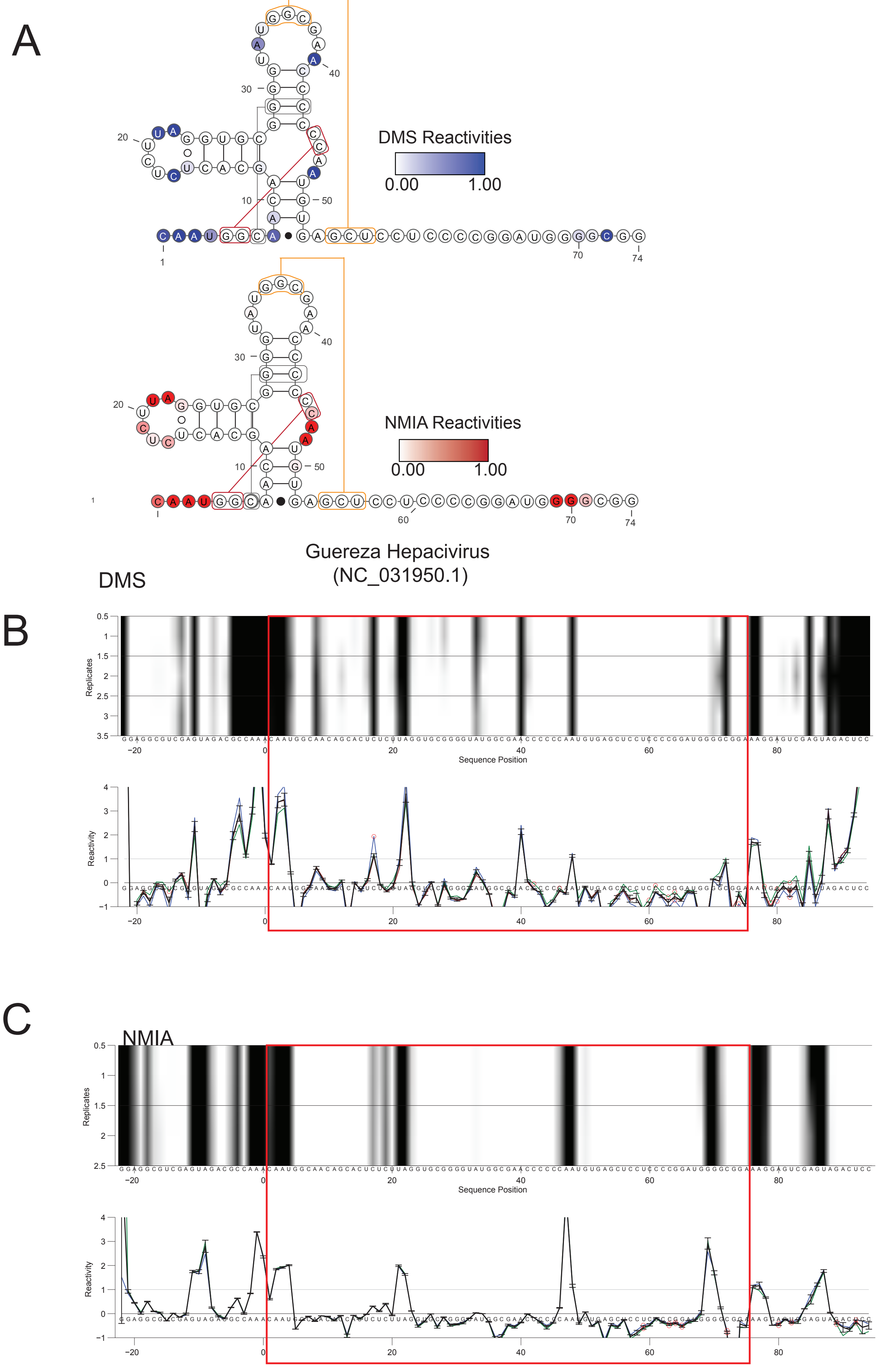

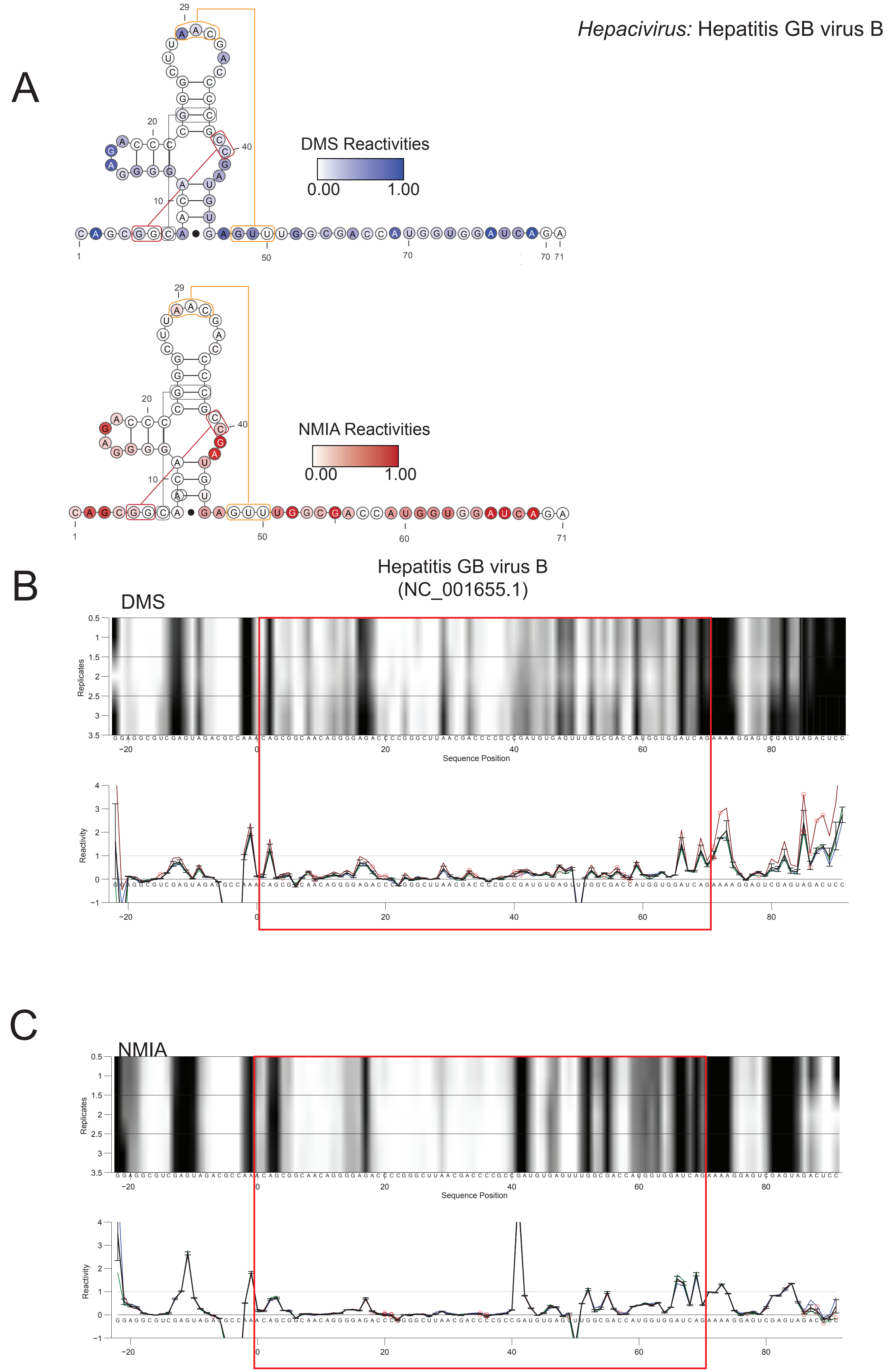

Hepacivirus: Hepatitis GB virus B
A
DMS Reactivities
0.00
1.00
NMIA Reactivities
0.00
1.00
B
Hepatitis GB virus B
(NC_001655.1)
DMS
Replicates
Sequence Position
Reactivity
C
NMIA
Replicates
Sequence Position
Reactivity
