# Supplementary material for: A New Subclass of Exoribonuclease-Resistant RNA Found in Multiple Genera of Flaviviridae
Source: mBio. 2020 Sep 29;11(5):e02352-20. doi: 10.1128/mBio.02352-20 (PMC7527734; doi:10.1128/mBio.02352-20)
Supplement: FIG S1 [file mBio.02352-20-sf001.pdf]

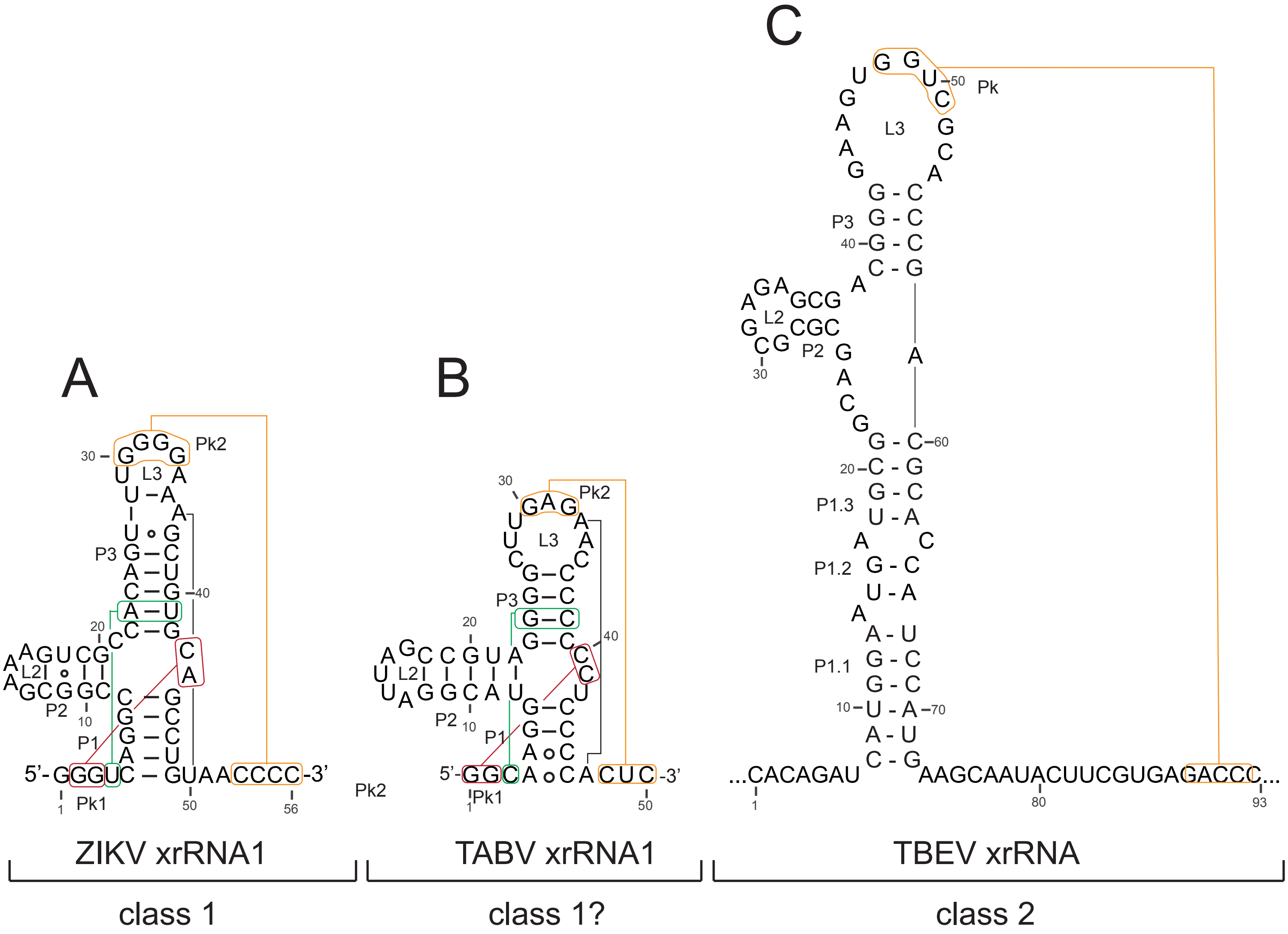
A
B
C
Pk2
Pk1
Pk
L2
L3
P1
P2
P3
P1.1
P1.2
P1.3
5'-
-3'
ZIKV xrRNA1
TABV xrRNA1
TBEV xrRNA
class 1
class 1?
class 2
